# Interaction of suppressor of cytokine signalling 3 with cavin-1 links SOCS3 function and cavin-1 stability

**DOI:** 10.1038/s41467-017-02585-y

**Published:** 2018-01-12

**Authors:** Jamie J. L. Williams, Nasser Alotaiq, William Mullen, Richard Burchmore, Libin Liu, George S. Baillie, Fred Schaper, Paul F. Pilch, Timothy M. Palmer

**Affiliations:** 1 School of Pharmacy and Medical Sciences, University of Bradford, Bradford, BD7 1DP UK; 2 Institute of Cardiovascular and Medical Sciences, University of Glasgow, Glasgow, G12 8QQ UK; 3 Polyomics Facility, University of Glasgow, Glasgow, G12 8QQ UK; 4 Departments of Biochemistry and Medicine, Boston University School of Medicine, Boston, MA 02118 USA; 5 Department of Systems Biology, Institute for Biology, Otto-von-Guericke-University Magdeburg, 39106 Magdeburg, Germany

## Abstract

Effective suppression of JAK–STAT signalling by the inducible inhibitor “suppressor of cytokine signalling 3” (SOCS3) is essential for limiting signalling from cytokine receptors. Here we show that cavin-1, a component of caveolae, is a functionally significant SOCS3-interacting protein. Biochemical and confocal imaging demonstrate that SOCS3 localisation to the plasma membrane requires cavin-1. SOCS3 is also critical for cavin-1 stabilisation, such that deletion of SOCS3 reduces the expression of cavin-1 and caveolin-1 proteins, thereby reducing caveola abundance in endothelial cells. Moreover, the interaction of cavin-1 and SOCS3 is essential for SOCS3 function, as loss of cavin-1 enhances cytokine-stimulated STAT3 phosphorylation and abolishes SOCS3-dependent inhibition of IL-6 signalling by cyclic AMP. Together, these findings reveal a new functionally important mechanism linking SOCS3-mediated inhibition of cytokine signalling to localisation at the plasma membrane via interaction with and stabilisation of cavin-1.

## Abstract (web summary)

SOCS3 is an important negative feedback inhibitor of JAK–STAT-mediated cytokine signalling. Here the authors implicate cavin-1 — an essential component of caveolae — in the recruitment of SOCS3 to the plasma membrane to prevent sustained cytokine receptor signalling.

## Introduction

Cytokines control many important biological responses, including haematopoiesis, T-cell differentiation and expansion, and inflammatory status^1,2^. Multiple temporally distinct inhibitory mechanisms operate to ensure signalling responses downstream of activated cytokine receptors are transient in nature. Therefore, sustained pathway activation perpetuates chronic inflammatory conditions such as rheumatoid arthritis and colitis, haematological malignancies such as polycythemia vera, and also solid tumour development^3–5^.

Several cytokine receptors, including gp130 (the signal transducing component of the interleukin-6 (IL-6) signalling complex), activate receptor-associated Janus kinases (JAKs) which then trigger receptor engagement with proteins such as signal transducer and activators of transcription (STATs), particularly STAT3. Phosphorylated STATs can then dimerise and translocate to the nucleus, where they function as transcription factors by binding to specific promoter elements and recruiting transcriptional co-activators^1,2^.

“Suppressors of cytokine signalling” (SOCS) proteins comprise a family of eight related members (cytokine-inducible SH2-containing protein (CIS), SOCS1–7) identified initially by their role as cytokine-inducible negative feedback inhibitors of signal propagation from specific cytokine receptors^6^. SOCS3 is recruited to activated cytokine receptors following the formation of a SOCS3 interaction motif upon phosphorylation of key Tyr residues by cytokine-activated JAKs. SOCS3 terminates signalling from gp130 by binding via a central SH2 domain to PTyr759, allowing it to interact with and inhibit adjacent receptor-bound JAKs via its kinase inhibitory region (KIR) thereby preventing the recruitment and tyrosine phosphorylation of STATs^7^. The C-terminal SOCS box domain directs SH2 domain-bound interacting proteins for ubiquitylation due to its ability to bind elongin B and C, Cullin family member Cul5, and RING (Really Interesting New Gene) finger protein Rbx2^7^. Following SOCS3-dependent ubiquitylation, targets such as FAK1 can be degraded either by the proteasome^8,9^ or, in the case of the granulocyte colony-stimulating factor receptor (G-CSFR), by trafficking into lysosomal compartments following internalisation^10^. However, despite advances in our molecular understanding of how SOCS3 interacts with cytokine receptors and JAKs, the extent to which other cellular proteins regulate SOCS3 function is unclear. Recently, CUE domain-containing 2 (CUEDC2) was identified as a novel SOCS3-interacting protein that could enhance its interaction with elongin C^11^. Such observations raise the possibility that additional protein interactors may be required to maximise the ability of SOCS3 to regulate signalling.

Cavin-1 (alternatively known as polymerase I and transcript release factor (PTRF)) is an abundant component of caveolae, which function as specialised lipid raft microdomains within the plasma membrane. Caveolae were first identified by electron microscopy as 50–100 nm flask-shaped plasma membrane invaginations^12^ and are now known to play critical roles in controlling endocytosis, sphingolipid metabolism, and compartmentalisation of signalling pathways^13^. Cavin-1, which is one of a family of four related proteins (cavins 1 to 4), is recruited by one or more “caveolin” proteins (caveolins 1 to 3) to the plasma membrane during the latter stages of caveola biogenesis, and is thought to be essential for caveola formation by stabilising caveolin proteins at the plasma membrane^14^.

While some studies have demonstrated localisation of cytokine receptors and JAKs in lipid raft microdomains^15–18^, little is known about the impact of caveolin expression/function on JAK–STAT signalling and no studies have specifically examined a role for cavins. In this study, we identify a novel interaction between SOCS3 and cavin-1. This interaction is not only required for optimal SOCS3-mediated inhibition of IL-6-mediated JAK–STAT signalling but also for effective stabilisation of cavin-1 and hence caveolin-1. Therefore, our findings define a new relationship between SOCS3 and cavin-1 in which each partner plays previously unappreciated roles in maintaining effective inhibition of JAK–STAT signalling (cavin-1), cavin-1 expression, and caveola stability (SOCS3).

## Results

### Cavin-1 as a SOCS3-regulated ubiquitylated protein

As well as inhibiting cytokine receptor signalling by inhibiting the Tyr kinase activity of receptor-bound JAKs^19^, SOCS3 can also control the stability of SH2 domain-bound proteins as part of an elongin/cullin/SOCS3 (ECS^SOCS3^) E3 ubiquitin ligase complex^6,7,20^. While several ubiquitinated substrates of SOCS3 are known, the full spectrum has yet to be identified. Thus, we pursued an experimental approach to elaborate on SOCS3 function by investigating SOCS3-regulated proteins. Since there is no consensus sequence for ubiquitylation, we used a stable isotopic labelling of amino acids in cell culture (SILAC)/mass spectrometry approach to compare ubiquitinomes from wild-type *SOCS3*^+/+^ (WT) and *SOCS3*^*−/−*^ murine embryonic fibroblast (MEF) cell lines expressing equivalent levels of a tandem affinity purification-compatible tagged ubiquitin transgene following SOCS3 induction (Supplementary Fig. 1). Using this approach, ubiquitylated proteins regulated by SOCS3 would be predicted to be enriched in WT but not *SOCS3*^*−/−*^ MEFs.

A ubiquitin transgene containing a tandem hexahistidine and biotin tag (HB-Ub) was used to allow two-step tandem affinity purification of the ubiquitinome via sequential Ni-NTA and streptavidin affinity chromatography under fully denaturing conditions necessary to inactivate deubiquitinases and prevent co-purification of non-covalently interacting ubiquitin-binding proteins^21,22^. Stable HB-Ub-expressing WT and *SOCS3*^*−/−*^ MEFs were generated via retrovirus-mediated gene transfer and assessed by immunoblotting for equivalent expression levels of the HB-Ub transgene. SOCS3 was induced by elevation of intracellular cyclic adenosine monophosphate (cAMP) levels with forskolin (50 μM), a direct activator of adenylyl cyclase. To increase the probability of detecting proteins whose ubiquitylation was SOCS3 dependent, protein tyrosine phosphatases were inhibited using a combination of sodium orthovanadate and H_2_O_2_ to preserve the PTyr status of potential substrates and maximise interaction with the SOCS3 SH2 domain^23^, while the ubiquitinome was preserved using proteasome inhibitor MG132. Tandem affinity-purified ubiquitinomes from WT and SOCS3^*−/−*^ MEFs were then analysed using an Orbitrap Velos FTMS and data processed using the MaxQuant computational platform^24^ (170). Under these conditions, MaxQuant detected cavin-1 (O54724) with a log_2_(normalised H L^−1^) = 1.37 (5 unique peptides, count ratio of 6). This suggested that cavin-1 was a ubiquitylated protein specifically depleted in SOCS3^*−/−*^ MEFs.

### SOCS3 enhances cavin-1 stability

**Fig. 1:**
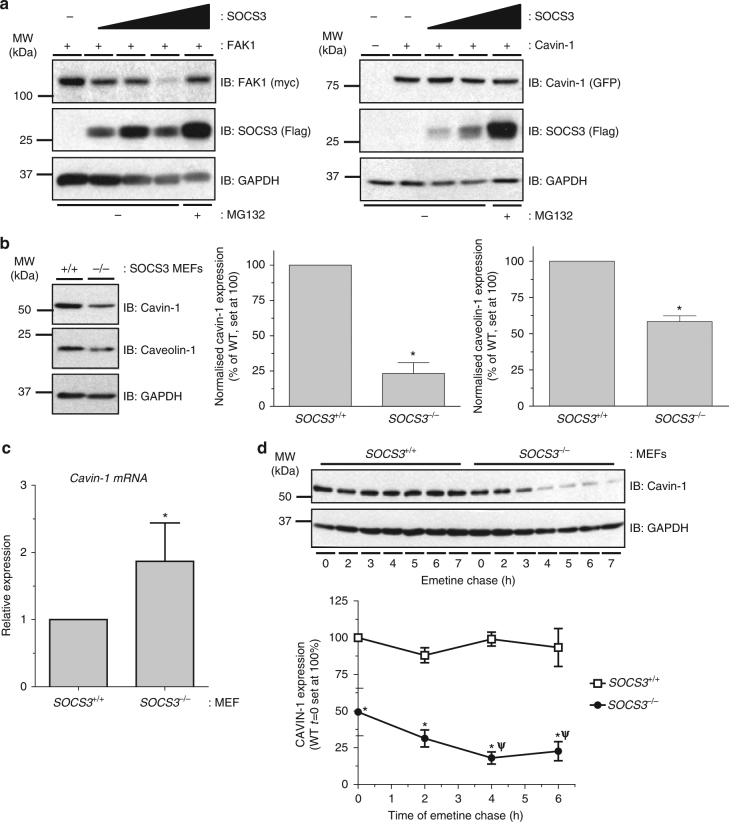
Cavin-1 stability is enhanced in the presence of SOCS3. **a** HEK293 cells co-transfected with fixed amounts of either myc-tagged FAK1 or GFP-cavin-1 expression constructs and increasing levels of Flag-SOCS3 were treated with or without proteasome inhibitor MG132 (6 μM) as indicated. Detergent-soluble whole-cell lysates were analysed by SDS-PAGE and immunoblotting. **b** Detergent-soluble whole-cell lysates prepared from *SOCS3*^*+/+*^ (+/+) and *SOCS3*^*−/−*^ (−/−) MEFs equalised for protein content were analysed by SDS-PAGE and immunoblotting. **c** Quantitative real-time PCR of cavin-1 mRNA levels in WT (*SOCS3*^*+/+*^) and *SOCS3*^*−/−*^ MEFs. Data are presented as mean ± standard error for *N* = 3 experiments. **d** Upper: Protein-equalised soluble cell extracts from *SOCS3*^*+/+*^ and *SOCS3*^*−/−*^ MEFs chased for the indicated times in serum-free medium with protein synthesis inhibitor emetine (100 μM) were analysed by SDS-PAGE and immunoblotting with the indicated antibodies. Lower: Quantitation of cavin-1 levels in *SOCS3*^*+/+*^ and *SOCS3*^*−/−*^ MEFs is also shown. Results are presented as mean values ± standard error for *N* = 3 experiments. *P < 0.05 vs. corresponding treatment in *SOCS3*^*+/+*^ MEFs, ^ψ^*P* < 0.05 vs. *t* = 0

Our proteomics screen suggested that SOCS3 regulates cavin-1 stability. To examine this, we compared the ability of increasing levels of SOCS3 to trigger the proteasomal degradation of co-transfected cavin-1 and FAK1, a previously characterised substrate of ECS^SOCS3^^8,9^. Consistent with previous work, increased SOCS3 expression triggered a decrease in FAK1 levels that could be rescued by inclusion of proteasome inhibitor MG132. In contrast, levels of cavin-1 were not altered even at the highest level of SOCS3 expression and were not increased by proteasome inhibitor MG132 (Fig. 1a). To determine whether SOCS3 could regulate levels of endogenous cavin-1, we assessed the effects of SOCS3 deletion on cavin-1 expression levels in MEFs. Immunoblotting of whole-cell extracts revealed that levels of cavin-1 protein were significantly reduced in *SOCS3*^*−/−*^ MEFs versus WT cells. This reduction occurred under conditions in which cavin-1 mRNA levels were significantly increased in *SOCS3*^*−/−*^ MEFs versus WT cells (Fig. 1b, c). The decrease in cavin-1 protein was paralleled by a similar decrease in caveolin-1 expression levels (Fig. 1b), which is consistent with previous studies showing that loss of cavin-1 triggers reductions in all three caveolin isoforms^14^. We then measured the half-lives of cavin-1 protein in WT and *SOCS3*^*−/−*^ MEFs by monitoring time-dependent changes in cavin-1 expression in whole-cell extracts following inhibition of protein synthesis with emetine^25^. For these experiments, cells were also pre-treated with forskolin (Fsk) to elevate cAMP levels and increase SOCS3 expression in WT MEFs^26,27^ Direct comparison of cavin-1 stability in WT versus *SOCS3*^*−/−*^ MEFs demonstrated that the absence of SOCS3 significantly reduced the half-life from >8 h (WT MEFs) to 2 h (*SOCS3*^*−/−*^ MEFs: Fig. 1d). Thus, in contrast to the well-defined role of SOCS3 in destabilising target proteins by targeting them for ubiquitylation and proteasomal degradation, the presence of SOCS3 stabilised cavin-1.

### Effect of SOCS3 deletion on caveola abundance

Our data thus far suggested that SOCS3 was an important regulator of caveolin-1 abundance via stabilisation of cavin-1. Homozygous deletion of the cavin-1 gene in mice results in marked reductions in the expression of all caveolin isoforms and a lack of detectable caveolae in multiple cell types, including endothelial cells (ECs) in which caveolae are especially abundant^14^.

**Fig. 2:**
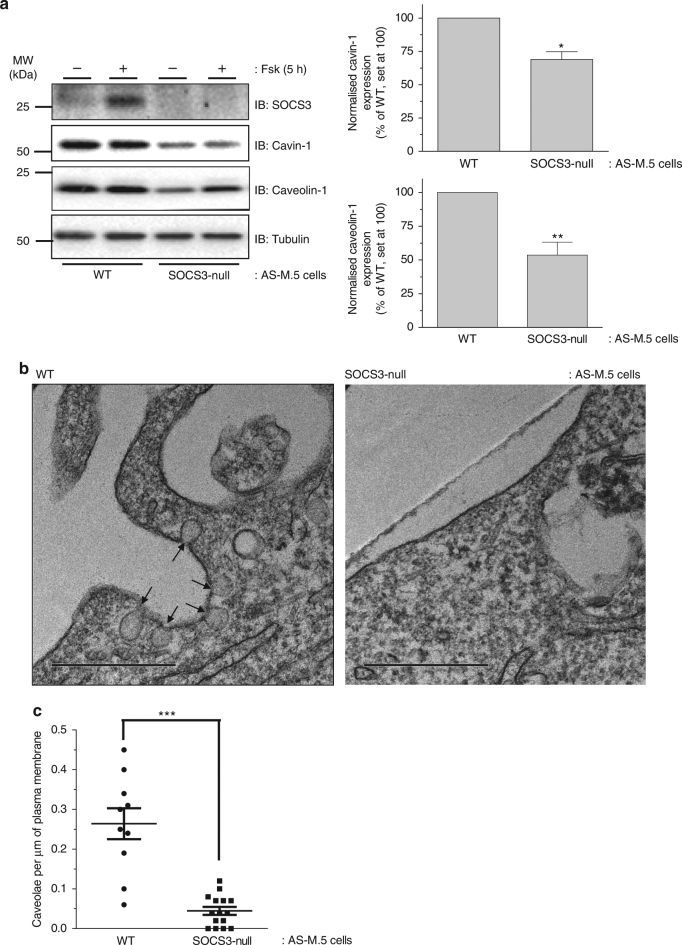
Effect of SOCS3 deletion on caveola abundance in endothelial cells. **a** Upper: Detergent-soluble whole-cell lysates from WT and SOCS3-null AS-M.5 human angiosarcoma-derived ECs treated with either vehicle or 50 μM Fsk for 5 h were equalised for protein content for SDS-PAGE for immunoblotting with the indicated antibodies. Lower*:* Quantitation of cavin-1 and caveolin-1 protein levels in unstimulated AS-M.5 cells is presented as mean ± standard error for *N* = 3 experiments. *P < 0.05, **P < 0.01 vs. WT cells. **b** Transmitting electron microscopy (TEM) was performed on WT and SOCS3-null AS-M.5 cells as indicated. Cell surface caveolae (indicated by the arrows) were readily detectable in WT cells (left panel). In contrast, plasma membranes from SOCS3-null cells were flat and caveolae density was significantly reduced compared to WT cells (right panel). Scale bar = 0.5 μm. **c** Quantitation of caveola density (number of caveolae per μm of plasma membrane) in WT and SOCS3-null AS-M.5 cells. ***P < 0.0001 vs. WT cells

To examine the impact of SOCS3 on caveola abundance, clustered regularly interspaced short palindromic repeats (CRISPR)/Cas9 technology was used to generate SOCS3-null AS-M.5 human angiosarcoma-derived immortalised ECs^28^. Treatment of WT AS-M.5 cells with cAMP-elevating agent Fsk was able to promote SOCS3 induction similar to that observed in MEFs and primary EC lines as previously reported^26,27^ However, this effect was lost in SOCS3-null AS-M.5 cells, while Nur77, a well-characterised cAMP-inducible gene product^29^, was detectable in both WT and SOCS3-null AS-M.5 cells following Fsk treatment (Fig. 2a, Supplementary Fig. 2). Similar to MEFs (Fig. 1b), SOCS3 deletion significantly reduced cavin-1 and caveolin-1 protein levels in AS-M.5 whole-cell extracts (Fig. 2a), demonstrating that this effect is independent of the cell system being investigated.

We then used transmission electron microscopy (TEM) to assess any consequences of the observed changes in cavin-1 and caveolin-1 expression on the abundance of cell surface caveolae. Caveolae were readily detectable in WT AS-M.5 cells as plasma membrane-localised flask-shaped invaginations ranging from 50 to 100 nm in diameter (Fig. 2b). In contrast, these were barely detectable in SOCS3-null cells (Fig. 2b, c). Therefore, significant reductions in cavin-1 and caveolin-1 protein levels triggered by the loss of SOCS3 in endothelial cells are translated into significantly reduced numbers of cell surface caveolae.

### Cavin-1 interacts with SOCS3 via a SH2 domain PEST sequence

**Fig. 3:**
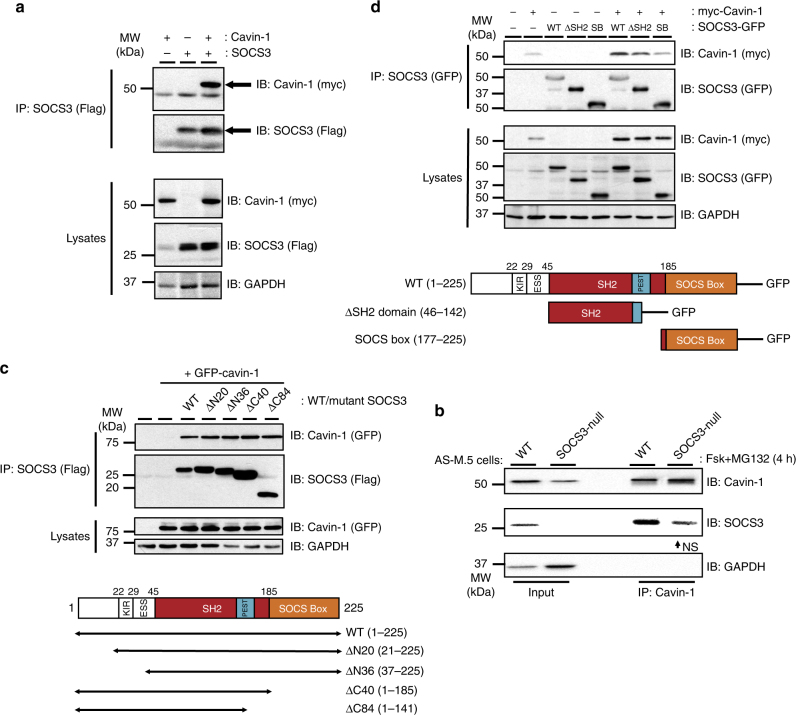
A region within the SOCS3 SH2 domain is necessary and sufficient for cavin-1 interaction. **a** Protein-equalised soluble cell extracts from HEK293 cells transfected with expression constructs encoding Flag-SOCS3 and myc-tagged cavin-1 as indicated were processed by immunoprecipitation (IP) with anti-Flag M2-agarose beads prior to SDS-PAGE and immunoblotting with the indicated antibodies. Whole-cell lysates from the samples used in the IP were also fractionated by SDS-PAGE for immunoblotting in parallel. **b** Protein-equalised soluble cell extracts from WT and SOCS3-null AS-M.5 cells treated with 50 μM Fsk and 6 μM MG132 for 4 h were processed by IP with anti-cavin-1 antibody prior to SDS-PAGE and immunoblotting with the indicated antibodies. As loss of SOCS3 in AS-M.5 cells reduces cavin-1 protein levels (**a**), twice the amount of protein was used as input for SOCS3-null cells to compensate. NS = non-specific band. **c** Upper: Protein-equalised soluble cell extracts from HEK293 cells transfected with expression constructs encoding either Flag-tagged WT SOCS3 or the indicated truncation mutants and GFP-tagged cavin-1 as indicated were processed by IP with anti-Flag M2-agarose beads prior to SDS-PAGE and immunoblotting with the indicated antibodies. Whole-cell lysates from the same samples used in the IP were also fractionated by SDS-PAGE for immunoblotting in parallel. Lower: Schematic of the SOCS3 truncation constructs used, KIR = kinase inhibitory region, ESS = extended SH2 subdomain, PEST = PEST (ProGluSerThr) motif. **d** Upper: Protein-equalised soluble cell extracts from HEK293 cells co-transfected with expression constructs encoding either full-length SOCS3 (WT), a truncated ΔSH2 SOCS3domain (ΔSH2), or SOCS box domain (SB) fused to GFP and myc-tagged cavin-1 as indicated were processed by IP with anti-GFP antibody and protein G-Sepharose beads prior to SDS-PAGE and immunoblotting with the indicated antibodies. Whole-cell lysates from the same samples used in the IP were also fractionated by SDS-PAGE and for immunoblotting in parallel. Lower: Schematic of the SOCS3-GFP fusions used

To assess whether SOCS3 could directly interact with cavin-1, co-immunoprecipitation (co-IP) experiments were performed in lysates isolated from transfected HEK293 cells transiently expressing Flag-SOCS3 and myc-cavin-1. These experiments demonstrated that myc-cavin-1 was present in anti-Flag antibody immunoprecipitates only when co-expressed with Flag-SOCS3, indicating the two proteins formed a complex (Fig. 3a). Similar results were obtained using Flag-cavin-1 and HA-SOCS3 (Supplementary Fig. 3), indicating that the effect was independent of the combination of tags used. Analysis of lysates and unbound samples from the experiments demonstrated that under conditions in which SOCS3 could be fully precipitated from lysates, a proportion of cavin-1 remained unbound, suggesting that not all available cavin-1 could interact with SOCS3 under these condition (Supplementary Fig. 4). To assess the interaction of endogenously expressed SOCS3 and cavin-1, WT and SOCS3-null AS-M.5 cells were stimulated with Fsk prior to IP of cavin-1 and analysis by immunoblotting. These experiments demonstrated that immunoreactive SOCS3 was specifically enriched in cavin-1 IPs from WT AS-M.5 cells (Fig. 3b), consistent with the co-IP data from experiments using transfected cells (Fig. 3a).

To identify the regions within SOCS3 that are important for SOCS3/cavin-1 interaction, we initially utilised a panel of Flag-tagged N- and C-terminal SOCS3 truncation mutants^30,31^ for their ability to co-IP green fluorescent protein (GFP)-tagged cavin-1^32^ as compared to WT SOCS3. Interestingly, all of the truncation mutants tested were able to co-IP GFP-cavin-1 to the same extent as full-length WT SOCS3 (Fig. 3c), suggesting that a region within the SH2 domain present in each of the mutants was necessary for SOCS3 binding to cavin-1. To test this, we expressed full-length SOCS3 (residues 1–225) and the region of the SOCS3 SH2 domain (residues 46–142, termed SOCS3 ΔSH2) required for cavin-1 binding (Fig. 3d) as GFP-tagged fusion proteins and compared their ability to co-IP myc-tagged cavin-1 in transfected HEK293 cells. As a negative control, we used a GFP fusion protein containing residues 177–225 of the SOCS box that we identified as dispensable for cavin-1 interaction (Fig. 3c). Similar to WT SOCS3-GFP, SOCS3 ΔSH2-GFP was able to co-IP myc-tagged cavin-1 above the non-specific levels observed with the SOCS3 SOCS box-GFP fusion and cavin-1 alone, albeit not to the same extent as WT SOCS3-GFP (Fig. 3d, lane 2 versus lane 8). Therefore, these data showed that residues 46–142 within the SOCS3 SH2 domain were both necessary and sufficient for SOCS3 interaction with cavin-1.

**Fig. 4:**
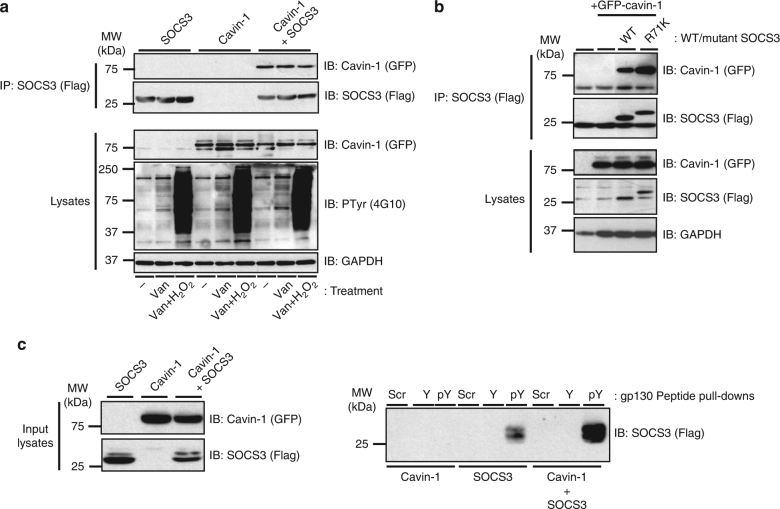
Cavin-1–SOCS3 interaction occurs independently of the PTyr binding capacity of the SH2 domain. **a** HEK293 cells transfected with expression constructs encoding Flag-SOCS3 and GFP-tagged cavin-1 as indicated were treated with or without Tyr phosphatase inhibitors sodium orthovanadate (Van: 1 mM) for 1.5 h and then hydrogen peroxide (H_2_O_2_: 0.2 mM) for an additional 30 min prior to harvesting. Protein-equalised soluble cell extracts were then processed by IP with anti-Flag M2-agarose beads prior to SDS-PAGE and immunoblotting with the indicated antibodies. Whole-cell lysates from the samples used in the IP were also fractionated by SDS-PAGE for immunoblotting in parallel. **b** Protein-equalised soluble cell extracts from HEK293 cells transfected with expression constructs encoding either WT or R71K-mutated Flag-SOCS3 and GFP-tagged-cavin-1 as indicated were processed by IP with anti-Flag M2-agarose beads prior to SDS-PAGE and immunoblotting with the indicated antibodies. Whole-cell lysates from the samples used in the IP were also fractionated by SDS-PAGE for immunoblotting in parallel. **c** Protein-equalised soluble cell extracts from HEK293 cells transfected with expression constructs encoding WT Flag-SOCS3 and GFP-tagged cavin-1 as indicated were incubated with 100 nM N-terminally biotinylated peptides corresponding to the Tyr759 motif of gp130 in its phosphorylated (pY) or non-phosphorylated (Y) forms or a scrambled control (Scr) and streptavidin–agarose beads prior to SDS-PAGE and immunoblotting with the indicated antibodies. Whole-cell lysates from the samples used in the pull-down were also fractionated by SDS-PAGE for immunoblotting in parallel

As many SOCS3 binding partners, including gp130, CD33 and FAK1, must be Tyr phosphorylated in order to interact with SOCS3, we pursued three experimental approaches to examine whether or not the PTyr-binding pocket within the SOCS3 SH2 domain was required for interaction with cavin-1. First, we treated transfected HEK293 cells with protein Tyr phosphatase inhibitor sodium orthovanadate in the presence or absence of hydrogen peroxide^23^. These experiments demonstrated that the isolation of GFP-cavin-1 in anti-Flag (SOCS3) immunoprecipitates was not altered by increases in global Tyr phosphorylation levels (Fig. 4a), suggesting that cavin-1 formed a complex with SOCS3 via a mechanism that did not require prior Tyr phosphorylation. Secondly, we tested R71K-mutated SOCS3, in which the conserved PTyr binding site within the SOCS3 SH2 domain is disrupted^30,31^, for its ability to form a complex with cavin-1. Co-IP assays revealed that a R71K-mutated SOCS3 bound cavin-1 equivalently to WT SOCS3 (Fig. 4b), again supporting the concept that cavin-1 interacted with the SOCS3 SH2 domain in a manner independent of its capacity to bind Tyr-phosphorylated ligands. Finally, N-terminally biotinylated peptides encompassing the Tyr759 motif of gp130 in phosphorylated (PTyr759 peptide) and non-phosphorylated (Tyr759 peptide) forms were used as bait to test the effect of cavin-1 co-expression on the ability of SOCS3 to be precipitated in peptide pull-down assays. As reported by others^30^, SOCS3 specifically associated with the PTyr759 peptide under these conditions. Using a maximally effective concentration of peptide (100 nM), co-expression with cavin-1 did not reduce the ability of SOCS3 to precipitate with PTyr759 peptide (Fig. 4c). Taken together, these data demonstrated that cavin-1 interacted with the SOCS3 SH2 domain at a location distinct from the well-defined PTyr-binding pocket.

**Fig. 5:**
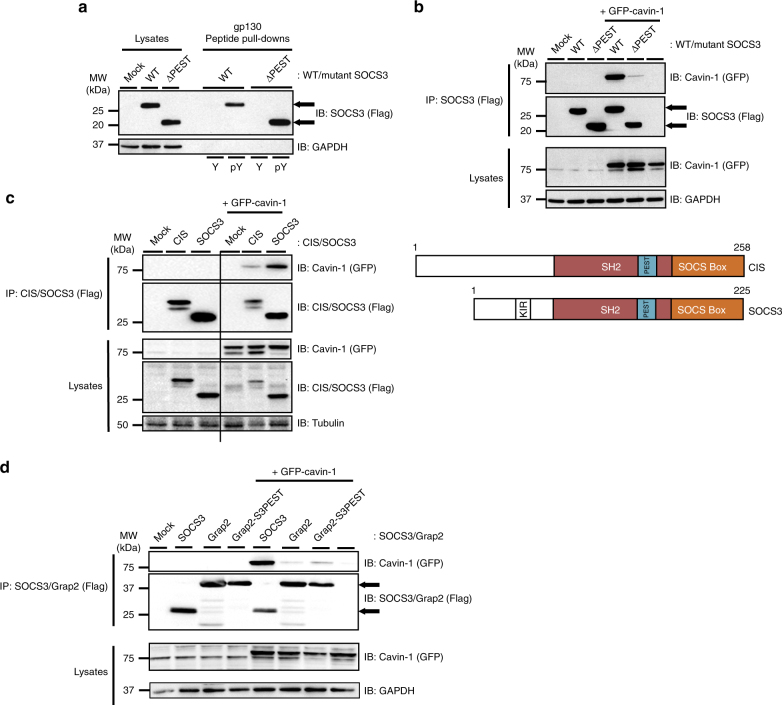
Cavin-1–SOCS3 interaction requires the SOCS3 SH2 PEST motif. **a** Protein-equalised soluble cell extracts from HEK293 cells transfected with expression constructs encoding either WT or ΔPEST Flag-SOCS3 were incubated with 100 nM N-terminally biotinylated peptides corresponding to the Tyr759 motif of gp130 in its phosphorylated (pY) or non-phosphorylated (Y) forms and streptavidin–agarose beads prior to loading with whole-cell lysate samples for SDS-PAGE and immunoblotting with the indicated antibodies. **b** Protein-equalised soluble cell extracts from HEK293 cells transfected with expression constructs encoding either WT or ΔPEST Flag-SOCS3 and GFP-tagged cavin-1 as indicated were processed by IP with anti-Flag M2-agarose beads prior to SDS-PAGE and immunoblotting with the indicated antibodies. Whole-cell lysates from the samples used in the IP were also fractionated by SDS-PAGE for immunoblotting in parallel. **c** Upper, Protein-equalised soluble cell extracts from HEK293 cells transfected with or without the indicated CIS and SOCS3 expression constructs and GFP-tagged cavin-1 as indicated were processed by IP with anti-Flag M2-agarose beads prior to SDS-PAGE and immunoblotting with the indicated antibodies. Whole-cell lysates from the samples used in the IP were also fractionated by SDS-PAGE for immunoblotting in parallel. Lower, Schematic of CIS and SOCS3. **d** Protein-equalised soluble cell extracts from HEK293 cells transfected with or without the indicated SOCS3 and Grap2 expression constructs and GFP-tagged cavin-1 as indicated were processed by IP with anti-Flag M2-agarose beads prior to SDS-PAGE and immunoblotting with the indicated antibodies. Whole-cell lysates from the samples used in the IP were also fractionated by SDS-PAGE for immunoblotting in parallel

The region of the SOCS3 SH2 domain identified in the studies above consists of two structurally distinct components. Firstly, residues 46–127 comprise of β-sheet and α-helical regions that form part of the PTyr-binding pocket common to all SH2 domains. Secondly, residues 128–142 form part of an unstructured PEST sequence insert that links the SH2 domain helix B with BG loop and βG strand motifs (residues 166–185)^33^. PEST motifs are unstructured hypermobile regions that have roles in multiple cellular processes by controlling protein–protein interactions and protein turnover^34,35^. We noted the presence of a PEST sequence within the classic SH2 domain structure is also displayed by CIS but none of the other SOCS family proteins^33^. Having excluded the PTyr-binding functionality for SH2 domain interaction with cavin-1, we next examined whether the PEST sequence insert was involved. To do this, we utilised a ΔPEST SOCS3 deletion mutant in which the PEST motif (Pro129-Arg163) was removed and replaced with (Gly-Ser)x4^36^. ΔPEST SOCS3 was expressed at comparable levels to WT SOCS3 in transfected HEK293 cells and, consistent with previously published work^33^, replacement of the PEST sequence did not diminish specific interaction with PTyr759 peptide as determined by in vitro peptide pull-down assays (Fig. 5a). In contrast, the ability of ΔPEST SOCS3 to bind cavin-1 in co-IP experiments was almost completely lost (Fig. 5b), thus demonstrating that the SOCS3 SH2 domain PEST insert was specifically required for cavin-1 interaction. Additional co-IP experiments using CIS, which also has a PEST insert in its SH2 domain, revealed that it was also able to form a complex with cavin-1 in co-transfected cells (Fig. 5c). Next we sought to determine whether the SOCS3 PEST sequence was sufficient to confer interaction with cavin-1. Bioinformatic analysis using ePESTfind (http://emboss.bioinformatics.nl/cgi-bin/emboss/epestfind) identified human Grap2 as a candidate SH2 domain-containing protein that lacked a detectable PEST sequence. In addition, Grap2 was unable to form a complex with cavin-1 upon co-expression in transfected HEK293 cells (Fig. 5d). Therefore, Pro129-Arg163 from SOCS3 was transplanted onto the central Grap2 SH2 domain and the resulting chimera (Grap2-S3PEST) assessed for its ability to form a complex with co-expressed cavin-1 in transfected HEK293 cells. These experiments demonstrated that insertion of SOCS3 PEST sequence was sufficient to confer an ability to bind cavin-1 on the resulting Grap2-S3PEST chimera, albeit to a much weaker extent than WT SOCS3 (Fig. 5d).

### Multiple cavin-1 regions required for SOCS3 interaction

**Fig. 6:**
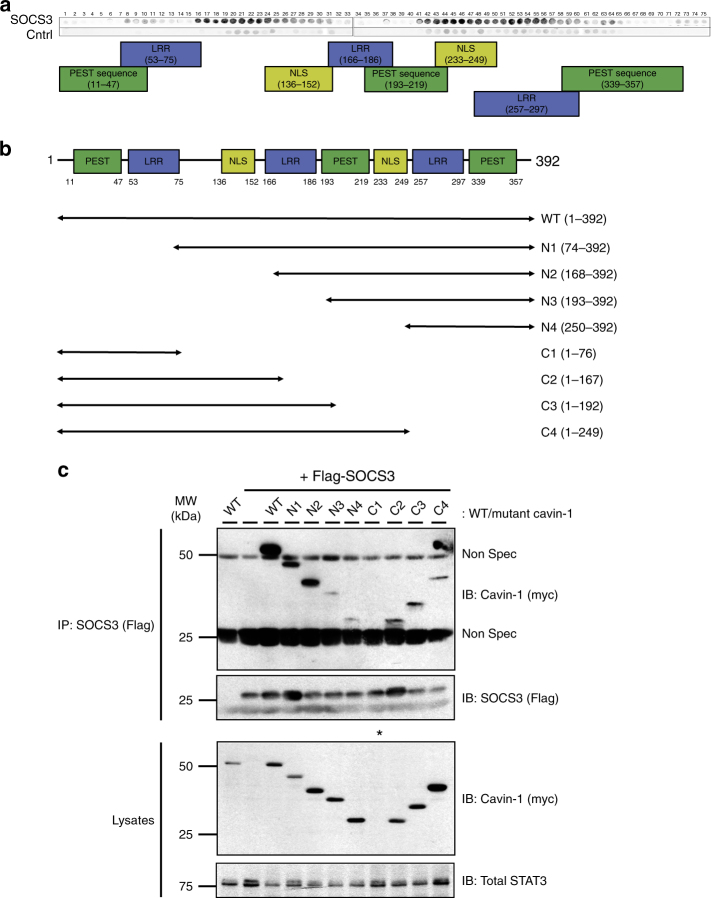
SOCS3 interacts with multiple regions within cavin-1. **a** An immobilised library of 25-mer peptides sequentially shifted by 5 amino acids along the entire cavin-1 open reading frame was overlaid with either purified SOCS3 or a negative control (Cntrl). Dark spots represent areas of interaction between SOCS3 and peptides within the cavin-1 peptide array. The domain structure of murine cavin-1 is indicated below the overlay. **b** Schematic representation of the N- and C-terminally truncated myc-tagged cavin-1 mutants used for co-IP experiments. **c** Protein-equalised soluble cell extracts from HEK293 cells transfected with expression constructs encoding either myc-tagged WT cavin-1 or the indicated truncation mutants and Flag-tagged SOCS3 as indicated were processed by IP with anti-Flag M2-agarose beads prior to SDS-PAGE and immunoblotting with the indicated antibodies. Whole-cell lysates from the same samples used in the IP were also fractionated by SDS-PAGE for immunoblotting in parallel. *Indicates that expression of the C1 cavin-1 mutant was not detectable; Non Spec refers to immunoglobulin-derived non-specific staining

To examine whether SOCS3 interacts directly with cavin-1, peptide arrays of overlapping 25-mer peptides sequentially shifted by five amino acids and spanning the full-length cavin-1 open reading frame were overlaid with purified recombinant SOCS3 and visualised by probing with anti-SOCS3 antibodies (Fig. 6a). Dark spots represent positive areas of SOCS3 interaction. Using this approach we found that SOCS3 could interact strongly with two distinct regions spanning >70 amino acids within the cavin-1 open reading frame: an N-terminal region spanning residues 75–152 and a C-terminal region encompassing residues 200–295. To validate the importance of these regions in controlling interaction with SOCS3 in intact cells, we generated a panel of myc epitope-tagged N- and C-terminal truncation mutants of cavin-1 (Fig. 6b) and tested their ability to co-IP Flag-SOCS3 upon co-expression in transfected HEK293 cells. All the truncated cavin-1 mutants expressed comparably to WT cavin-1 except for the C1 construct encoding residues 1–75 (Fig. 6c). These experiments demonstrated that, compared with WT cavin-1, each of the N-terminal and C-terminal truncation mutants was compromised in its capacity to co-IP with SOCS3. In conjunction with data from the peptide array experiments, our findings demonstrate that multiple SOCS3 binding interfaces within cavin-1 were required for optimal interaction with SOCS3.

### Cavin-1 promotes SOCS3 localisation to the plasma membrane

**Fig. 7:**
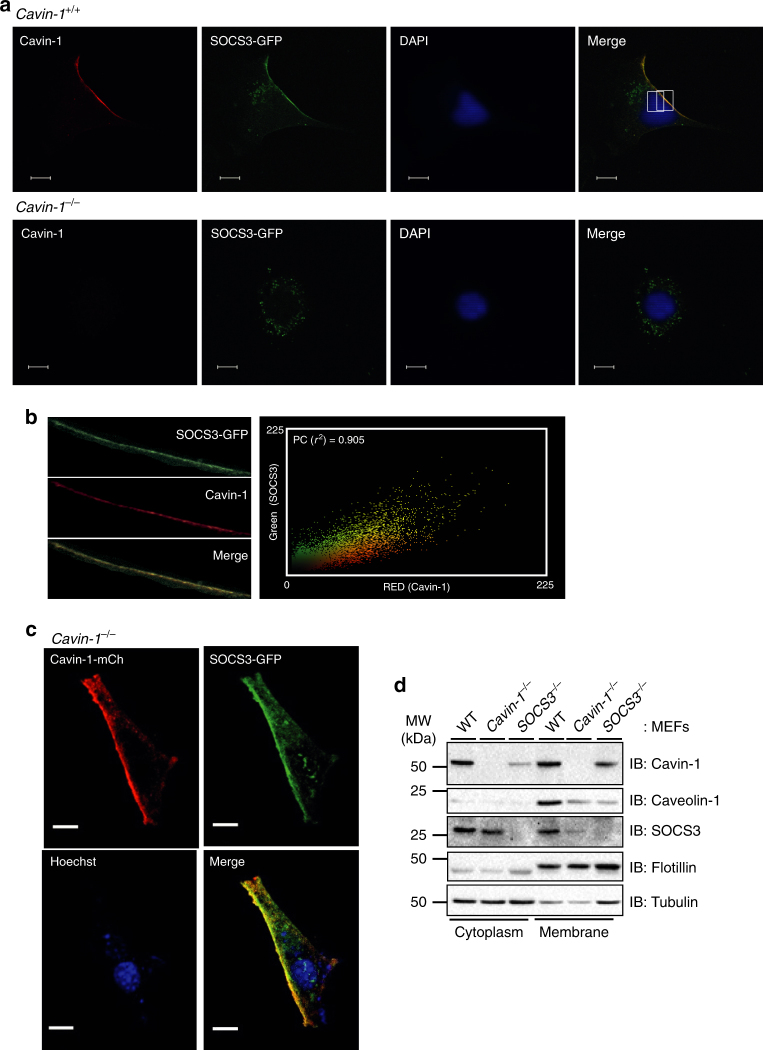
Cavin-1 drives SOCS3 localisation to the plasma membrane. **a** WT (*cavin-1*^*+/+*^) and *cavin-1*^*−/−*^ MEFs transiently expressing SOCS3-GFP (green) were stained with DAPI prior to being fixed, solubilised, and stained with anti-cavin-1 antibody (red) before mounting for imaging by confocal microscopy. Areas of red and green overlap are yellow. Scale bar = 10 μm. **b** Pearson's correlation coefficient value (*r*^2^) measured from the intensity values located within the rectangular region on the plasma membrane superimposed on the merged cavin-1/SOCS3-GFP image from WT MEFs. **c** *Cavin-1*^*−/−*^ MEFs transiently co-expressing SOCS3-GFP (green) and cavin-1-mCh (red) were stained with Hoechst 33342 prior to being fixed for imaging by confocal microscopy. Areas of red and green overlap are yellow. Scale bar = 10 μm. **d** WT, *SOCS3*^*−/−*^, and *cavin-1*^*−/−*^ MEFs were pre-incubated with Fsk (50 μM) for 5 h to induce SOCS3 prior to subcellular fractionation and analysis by SDS-PAGE and immunoblotting with the indicated antibodies

Cavin-1 is required for stabilisation and maturation of caveolae at the plasma membrane, although it is also present with caveolin-1 in non-lipid raft fractions^37^. SOCS3 is thought to be recruited to activated cytokine receptors at the plasma membrane following the formation of a SOCS3 interaction motif upon phosphorylation of key Tyr residues by cytokine-activated JAKs^19^. Therefore, to examine a role for cavin-1 in controlling SOCS3 localisation, we used confocal microscopy to assess the effect of cavin-1 deletion on the subcellular distribution of a SOCS3-GFP fusion protein expressed in transfected cells plated at low density. A transfected SOCS3-GFP construct was used for these experiments as we failed to specifically detect endogenous SOCS3 staining in WT MEFs over and above background staining in *SOCS3*^*−/−*^ MEFs in confocal imaging experiments using three separate commercially available antibodies. In transfected WT MEFs, two populations of SOCS3-GFP-derived fluorescence were detectable: a punctate intracellular pool and a plasma membrane-localised pool (Fig. 7a). Endogenous cavin-1 was localised predominantly at the plasma membrane of the trailing edge of the cells as described by others^38^. Merging of the images revealed co-localisation of SOCS3-GFP and cavin-1 specifically at the plasma membrane (Fig. 7a). Analysis of SOCS3-GFP/cavin-1 staining produced Pearson's correlation coefficient values of >0.90 at the plasma membrane, indicative of a high degree of co-localisation (Fig. 7b). Conversely, in *cavin-1*^*−/−*^ MEFs SOCS3-GFP was undetectable at the plasma membrane and only present within a punctate intracellular pool (Fig. 7a). Importantly, transient co-expression of SOCS3-GFP with co-transfected cavin-1-mCherry into *cavin-1*^*−/−*^ MEFs was able to restore their co-localisation at the plasma membrane (Fig. 7c). Expression of GFP alone in WT MEFs did not produce any detectable co-localisation with cavin-1, and its distribution was similar in both WT and *cavin-1*^*−/−*^ MEFs (Supplementary Fig. 5A, B).

Additionally, subcellular fractionation experiments demonstrated that cavin-1 was mainly present in membrane and cytoplasmic fractions. This mirrored the subcellular distribution of SOCS3 in WT MEFs following induction by Fsk treatment for 5 h. Interestingly, cavin-1 deletion shifted the distribution of SOCS3 predominantly to the cytoplasm (Fig. 7d). Thus, the presence of cavin-1 was important for localising endogenous SOCS3 to the membrane fraction, consistent with our confocal imaging experiments using SOCS3-GFP (Fig. 7a–c). Subcellular fractionation experiments also demonstrated that SOCS3 deletion produced a comparable reduction in caveolin-1 expression at the membrane as deletion of cavin-1, indicative of an indirect role for SOCS3 in maintaining caveolin-1 expression via stabilisation of cavin-1 (Fig. 7d). This change was specific for caveolin-1 as levels of the membrane marker flotillin were unaffected by deletion of either SOCS3 or cavin-1 (Fig. 7d). Therefore, together these data indicate that cavin-1 co-localised with a plasma membrane pool of SOCS3 in intact cells and was an important determinant of SOCS3 localisation to the plasma membrane.

### Cavin-1 limits IL-6-stimulated Tyr705 STAT3 phosphorylation

**Fig. 8:**
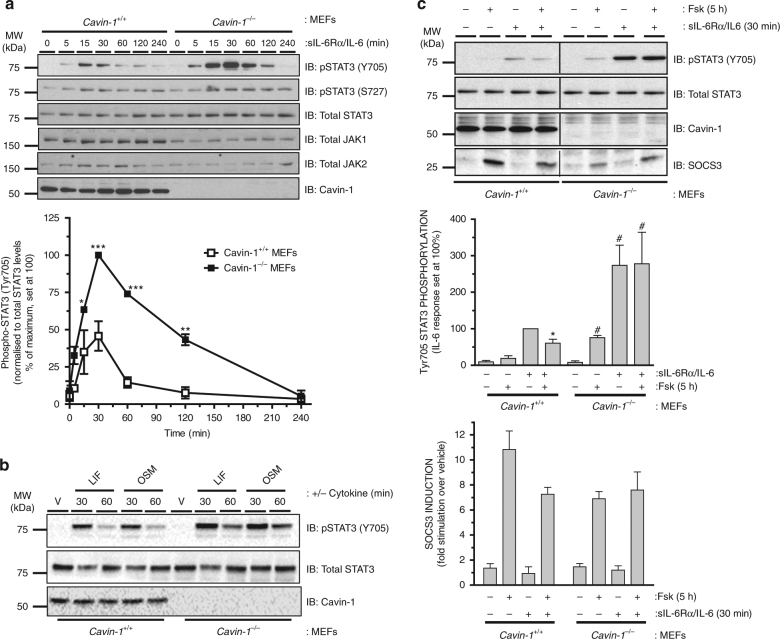
Cavin-1 limits Tyr705 phosphorylation of STAT3 via SOCS3. **a** Upper: Protein-equalised soluble cell extracts from *cavin-1*^*+/+*^ and *cavin-1*^*−/−*^ MEFs treated for the indicated times with sIL-6Rα/IL-6 (25 and 5 ng ml^−1^) were fractionated by SDS-PAGE prior to immunoblotting with the indicated antibodies. Quantitation of normalised Tyr705 phospho-STAT3 in *cavin-1*^*+/+*^ and *cavin-1*^*−/−*^ MEFs are presented as mean values ± standard error for *N* = 3 experiments. *P < 0.05, **P < 0.01, ***P < 0.001 vs. corresponding treatment in *cavin-1*^*+/+*^ MEFs. **b** Protein-equalised soluble cell extracts from *cavin-1*^*+/+*^ and *cavin-1*^*−/−*^ MEFs treated for 30 and 60 min with leukaemia inhibitory factor (LIF; 0.5 nM), oncostatin M (OSM; 10 ng ml^−1^) or vehicle (V) were fractionated by SDS-PAGE prior to immunoblotting with the indicated antibodies. **c** Upper: WT (*cavin-1*^*+/+*^) and *cavin-1*^*−/−*^ MEFs were pre-incubated with or without Fsk (50 μM) for 5 h prior to treatment with or without sIL-Rα/IL-6 (25 and 5 ng ml^−1^) for 30 min. Cell extracts were analysed by SDS-PAGE and immunoblotting with the indicated antibodies. Lower: Quantitation of normalised Tyr705 phospho-STAT3 and SOCS3 in *cavin-1*^*+/+*^ and *cavin-1*^*−/−*^ MEFs is presented as mean ± standard error for *N* = 3 experiments, *P < 0.05 vs. sIL-6Rα/IL-6 treatment in *cavin-1*^*+/+*^ MEFs, ^#^*P* < 0.05 vs. vehicle treatment in *cavin-1*^*−/−*^ MEFs.

While some studies have demonstrated localisation of cytokine receptors and JAKs in lipid raft microdomains^15–18^, relatively little is known about the impact of caveolin expression/function on JAK–STAT signalling and no studies have specifically examined a role for cavins. Our data suggested that cavin-1 and SOCS3 interacted directly and co-localised at the plasma membrane, while SOCS3 was mainly cytosolic in the absence of cavin-1. To examine any functional impact of cavin-1 on cytokine signalling, we examined the effects of cavin-1 deletion in MEFs on IL-6-mediated activation of STAT3, as determined by phosphorylation at Tyr705 which is required for STAT3 to form transcriptionally active complexes^39^. While stimulation of both WT and *cavin-1*^*−/−*^ MEFs with a sIL-6Rα/IL-6 *trans*-signalling complex triggered a transient increase in STAT3 phosphorylation on Tyr705, the response was greater and more sustained in *cavin-1*^*−/−*^ MEFs, being detectable at the 60 and 120 min time points in *cavin-1*^*−/−*^ but less pronounced in WT cells (Fig. 8a). Interestingly, Tyr705 phosphorylation was specifically enhanced as STAT3 phosphorylation on Ser727 (which is mediated by several candidate Ser/Thr kinases^40^) was unaffected by cavin-1 deletion. Moreover, the increase in IL-6 signalling occurred despite reduced levels of JAK1 in *cavin-1*^*−/−*^ MEFs, although this reduction did not reach statistical significance (Fig. 8a). Other cytokine receptor complexes that utilise gp130 include those for leukaemia inhibitory factor (LIF) and oncostatin M (OSM): LIF signals via gp130/LIF receptor (LIFR) heterodimers, while OSM signals downstream using either LIFR/gp130 or OSM receptor/gp130 complexes^41^. As observed with sIL-6Rα/IL-6, Tyr705 phosphorylation of STAT3 in response to either LIF or OSM was greater in *cavin-1*^*−/−*^ versus WT MEFs at 60 min (Fig. 8b). Taken together, these data suggested that loss of cavin-1 compromised one or more inhibitory mechanisms responsible for suppressing gp130- and JAK-mediated Tyr phosphorylation of STAT3.

Previous studies have demonstrated that depletion or loss of SOCS3 results in prolonged activation of STAT3 in response to specific cytokines^42–44^, similar to the effect observed upon cavin-1 deletion. We have shown previously that the ability of cAMP to inhibit IL-6 signalling in vascular ECs, MEFs, and COS cells has an absolute requirement for Epac1-dependent induction of SOCS3^26,27,45^. Given the importance of cavin-1 in localising SOCS3 to the plasma membrane and the sustained phosphorylation of STAT3 on Tyr705 observed following sIL-6Rα/IL-6 stimulation of *cavin-1*^*−/−*^ MEFs, we examined the impact of cavin-1 deletion on the inhibitory effect of cAMP which has previously been shown to be SOCS3 dependent^26,45^. These experiments demonstrated that while pre-treatment of WT MEFs with cAMP-elevating drug Fsk (50 μM) significantly inhibited sIL-6Rα/IL-6-stimulated Tyr705 phosphorylation of STAT3, this effect was lost in *cavin-1*^*−/−*^ MEFs even though Fsk in combination with sIL-6Rα/IL-6 produced equivalent levels of SOCS3 in WT and *cavin-1*^*−/−*^ MEFs (Fig. 8c). These results also did not reflect a non-specific reduction in cAMP responsiveness following loss of cavin-1 as Fsk could trigger the accumulation of cAMP target gene Nur77 equivalently in both WT and *cavin-1*^*−/−*^ MEFs (Supplementary Fig. 6). Therefore, the presence of cavin-1 was necessary for SOCS3-mediated inhibition of IL-6 signalling by cAMP.

## Discussion

The importance of SOCS3 in limiting downstream signalling from cytokine receptor complexes that utilise gp130, as well as the leptin receptor ObRb and the G-CSFR, is well established^6,7^ However, relatively little is known about how SOCS3 interaction with other intracellular proteins can impact on its ability to inhibit signalling. As part of a study to identify SOCS3-regulated substrates, we performed “stable isotopic labelling of amino acids in cell culture” (SILAC) analysis of ubiquitinome profiles in WT and *SOCS3*^*−/−*^ MEFs stably expressing a tandem affinity purification (TAP)-tagged ubiquitin transgene^46^. Using this approach, the caveola scaffolding protein cavin-1 was identified as a ubiquitinated protein whose levels were stabilised in WT cells. We have demonstrated that cavin-1 can interact with SOCS3 and that the absence of SOCS3 results in increased turnover of cavin-1 and a parallel reduction in cellular levels of caveolin-1 and cell surface caveloae. We have also demonstrated that cavin-1 is important for effective SOCS3-mediated suppression of JAK–STAT signalling in response to sIL-6Rα/IL-6 *trans*-signalling complexes.

The importance of caveolae and other lipid raft microdomains for maintaining signalling from the plasma membrane has been demonstrated for a variety of systems, including endothelial nitric oxide synthase and Src^47,48^. In comparison, relatively little information is available on how they regulate JAK–STAT pathway activation. Localisation of JAK–STAT signalling components, including gp130, receptors for growth hormone, ciliary neurotrophic factor and LIF, and JAK2 to lipid rafts has been determined by biochemical fractionation of cell extracts^15–18,49–52^. However, the functional consequences appear to be context dependent, such that raft disruption by treatment with cholesterol-depleting agents like β-cyclodextrin or homozygous deletion of caveolin-1 can either inhibit^15,16,49^ or enhance^52,53^ downstream signalling. Thus, Lisanti and colleagues^52^ have examined the effects of manipulating caveolin-1, and demonstrated that caveolin-1 can suppress prolactin receptor-mediated JAK2-dependent phosphorylation and activation of STAT5a in murine mammary epithelial cells in vitro, consistent with observations that caveolin-1 deletion in vivo enhances prolactin receptor signalling^53^. The mechanism proposed was via a direct interaction between caveolin-1 and JAK2, although no evidence of a direct effect of caveolin-1 on JAK2 Tyr kinase activity was presented^52^. Other studies have specifically examined the importance of caveolae for gp130 function, demonstrating that a significant proportion of cellular gp130 resides in detergent-resistant lipid rafts and can co-IP with caveolin-1. In addition, cholesterol depletion with β-cyclodextrin has been shown to trigger the re-distribution of gp130 to non-raft factions and attenuate the ability of IL-6 to stimulate STAT3 phosphorylation on Tyr705^16^. In contrast, others have found that both gp130 and STAT3 are localised to lipid rafts^15^ and demonstrated an inverse relationship between caveolin-1 expression and STAT3 activation^54^. Therefore, while a weak association between membrane microdomains and JAK–STAT signalling modules has been made, the molecular mechanisms responsible for this interaction remain unclear.

**Fig. 9:**
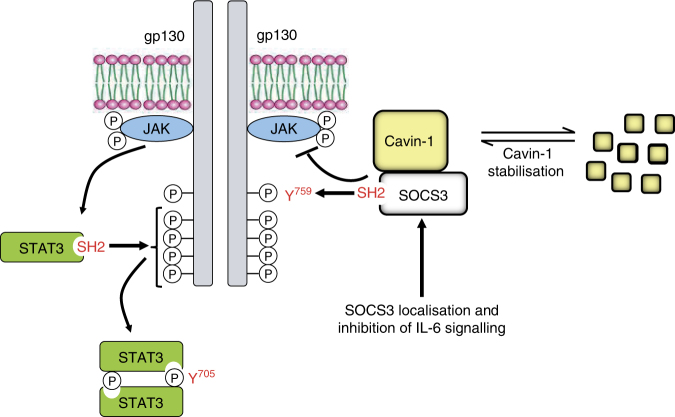
Model of novel functional interactions between SOCS3 and cavin-1

Our data would suggest a novel route through which caveola accessory protein cavin-1 can modulate cytokine receptor signalling via interaction with the inhibitory regulator SOCS3. While SOCS3 expression is induced in response to many stimuli, conditional gene targeting strategies have revealed that sensitivity to SOCS3 is restricted to a panel of plasma membrane-localised cytokine receptors^6,7,41^. Consistent with another study^38^, we found that while cavin-1 was localised to the plasma membrane in WT MEFs, it was not distributed uniformly, instead localising to the trailing edge of migrating cells. Importantly, a significant proportion of SOCS3-GFP co-localised to the same plasma membrane compartment in WT but not *cavin-1*^*−/−*^ MEFs. Together with data showing that cavin-1 could co-IP with SOCS3 and that purified SOCS3 could interact with multiple cavin-1-derived peptides in vitro, we propose that cavin-1 binds SOCS3 directly and that this contributes to efficient SOCS3 recruitment to the plasma membrane where it can effectively bind and inhibit cytokine receptors such as gp130. A key aspect of this model (Fig. 9) is that SOCS3 can still bind Tyr phosphorylated peptide in vitro in the presence of cavin-1. Interestingly, the SOCS3 SH2 domain appeared to fulfil both PTyr and cavin-1 binding functions as cavin-1 interaction required the PEST motif present within the SOCS3 SH2 domain, which we and others have shown to be dispensable for PTyr binding^33,36^. In some respects, this is similar to the recently described interaction between SOCS3 and CUEDC2, which also binds the SH2 domain and enhances SOCS3-mediated inhibition of JAK1–STAT3 activation by IL-6^11^. Since CUEDC2 potentiates SOCS3 function it would be anticipated that, like cavin-1, its interaction with the SH2 domain must be independent of PTyr binding, suggesting it may also involve the PEST sequence. However, in contrast to cavin-1, CUEDC2 localises to the cytoplasm and nucleus^55^. Nevertheless, our observations and those of Zhang et al.^11^ raise the possibility that multiple proteins may bind within the SOCS3 SH2 domain to facilitate localisation with Tyr phosphorylated binding partners in distinct subcellular compartments. In this regard, it should be noted that confocal imaging and subcellular fraction experiments detected SOCS3 in the cytoplasm as well as the plasma membrane, and that cavin-1 deletion resulted in the specific loss of the plasma membrane pool.

To date, we are only aware of one other study which has examined the impact of the PEST sequence on SOCS3 function^36^. However, these experiments were performed in HEK293 cells co-transfected to express a STAT3-responsive reporter gene and increasing amounts of either WT or ΔPEST SOCS3. The authors noted that at maximal levels of WT and ΔPEST SOCS3 expression, both constructs abolished LIF-stimulated activation of STAT3. However, upon normalising SOCS3 function with the expression levels of WT and ΔPEST SOCS3, they also noted that at submaximal expression levels the functionality of ΔPEST SOCS3 was less than that of WT SOCS3. Thus, they concluded that WT SOCS3 is slightly more efficient at inhibiting STAT3 activation^36^. Others have shown that low expression levels of SOCS3 inhibit signalling via interaction with g130 followed by inhibition of JAK activity, whereas overexpression SOCS3 can inhibit gp130 signalling independently of interaction with the SOCS3 binding site and works instead via direct inhibition of JAK1^19,56^ These data would also suggest that any functional deficits in ΔPEST SOCS3 in localising to gp130 would be overcome by its overexpression. In contrast, our functional experiments examining signalling from endogenous proteins suggest an important aspect of SOCS3 PEST motif function is an interaction with cavin-1 that is critical for effective regulation of JAK–STAT signalling. The effects on signalling of reconstituting *cavin-1*^*−/−*^ MEFs with mutated cavin-1 that fails to interact with SOCS3 but retains the ability to stabilise caveolin-1 would be very informative in dissecting whether cavin-1 is essential for SOCS3 function or simply enhances it through facilitating recruitment to the plasma membrane. It would also be important to assess any functional consequences of SOCS3 on gp130 ubiquitylation^57^ and receptor trafficking^10^ in order to fully assess the impact of cavin-1 on SOCS3 function.

While this adds an extra layer of regulation for SOCS3, our study has also identified a previously unknown mechanism by which SOCS3 can regulate cavin-1 function by enhancing its stability and, as a consequence, maintaining expression levels of caveolin-1 and cell surface caveolae. Similar observations have recently been reported for Eps15 homology domain-containing protein 2 (EHD2) which, to our knowledge, is the only other example of a cavin-1-interacting protein that regulates caveola stability, although a direct effect on cavin-1 turnover has not been examined^58^. More generally, our findings also raise the possibility that cavins constitute a new class of SOCS3-interacting proteins. While the presence of cavin-1 and caveolin-1 is sufficient to generate caveolae in many cell types^59^, MEFs also express cavin-2 and cavin-3. Elegant biochemical and biophysical studies have demonstrated that cavins assemble into oligomeric complexes both in cells and in vitro^60,61^. While each of the cavins is detectable on individual caveolae^59^, cavin-2 and cavin-3 appear to form distinct hetero-oligomeric complexes with cavin-1 rather than with each other^60^. Thus, it would be anticipated that SOCS3 should interact with both cavin-1/cavin-2 and cavin-1/cavin-3 oligomers and therefore distribute itself uniformly around caveolar bulbs similarly to cavin-1^61^. As the SOCS3 PEST sequence was necessary for cavin-1 interaction, it would also be informative to assess what extent this property is shared among similar sequences present in other SOCS family members. Analysis of the SOCS family revealed that CIS, which like SOCS3 can also interact with cavin-1, contains a PEST motif located in its SH2 domain, while SOCS1, SOCS5, and SOCS7 each have one or more PEST sequences located within their N-terminal domains. In contrast, no PEST motifs are found in SOCS2, SOCS4, and SOCS6. Given the distinct roles of different SOCS family members in regulating signalling^62^, the functional significance of the identified PEST motifs and their roles in determining the localisation of individual SOCS proteins via distinct protein interactions warrant further investigation.

Finally, our findings may have implications in the context of how cavin-1 and SOCS3 dysfunction can trigger disease. Several inactivating frameshift mutations within exon 2 of the cavin-1 gene that result in the production of altered cavin-1 proteins have been identified in patients with general lipodystrophy, muscular dystrophy, and insulin resistance^63–66^. In each case, a lack of functional cavin-1 is associated with the downregulation and/or mislocalisation of all three caveolin subtypes in skeletal muscle and an absence of cell surface caveolae in patient-derived fibroblasts^64^ and skeletal muscle^63^. As multiple regions within cavin-1 are required for optimal binding of SOCS3, the mutated cavin-1 proteins identified in patients with congenital generalised lipodystrophy would be predicted to be compromised in their ability to interact with SOCS3, thereby resulting in enhanced IL-6 responses. In this regard, cardiac-specific homozygous deletion of murine *SOCS3* results in contractile dysfunction and the occurrence of a variety of ventricular arrhythmias^67^, the latter of which is also observed in patients with inactivating cavin-1 mutations^64^. Finally, a F136L germline SOCS3 mutation found in a subset of polycythemia vera patients has been shown to display an impaired capacity to inhibit erythropoietin receptor-JAK2 signalling^68^. As F136 is located within the PEST insert we have identified as critical for cavin-1 interaction, this mutation may alter cavin-1 binding to SOCS3 to block its inhibitory effects on JAK–STAT signalling. Based on our findings, future studies will therefore need to examine how cavin-1 and/or SOCS3 mutations identified in patients interact to trigger defective regulation of signalling in these pathologies.

## Methods

### Cell culture and transfection

HEK293 cells were obtained from the European Collection of Authenticated Cell Cultures (ECACC) through Sigma. Immortalised *SOCS3*^*−/−*^ and *cavin-1*^*−/−*^ MEFs and the corresponding WT cell lines have been described previously^59,69^. HEK293 cells and MEFs were maintained in Dulbecco’s modified Eagle’s medium (DMEM) supplemented with 10% (v/v) foetal bovine serum (FBS), 1 mM l-glutamine, 100 U ml^−1^ penicillin and 100 μM streptomycin. AS-M.5 human angiosarcoma-derived ECs generously provided by Dr Vera Krump-Konvalinkova and Professor Charles Kirkpatrick (Johannes Gutenberg University of Mainz, Germany)^28^ were cultured in endothelial growth medium-2 supplemented with 2% (w/v) FBS, hydrocortisone, ascorbate, and recombinant growth factors as recommended by the supplier (Lonza). HEK293 cells at 80% confluence on poly-d-lysine-coated dishes were transfected with 2–8 μg of complementary DNA (cDNA) per 100 mm dish using PolyFect transfection reagent (Qiagen) as per THE manufacturer’s instructions.

For SILAC experiments, MEFs were grown in either heavy SILAC DMEM (^13^C_6_-arginine, ^13^C^6^-lysine; R6K6) or control SILAC DMEM (^12^C_6_-arginine, ^12^C^6^-lysine; R0K0) (Dundee Cell Products, UK) supplemented with 10% (v/v) dialysed calf serum, 100 U ml^−1^ penicillin, 100 μM streptomycin, 4 μg ml^−1^ puromycin, 200 mg l^−1^ l-proline and 1 μM d-biotin. Arginine can be metabolised from ^13^C_6_-arginine to an isotope of the non-essential amino acid ^13^C_5_-proline via the arginase pathway thus complicating data analysis^70^. As such, media were supplemented with l-proline (200 mg l^−1^) to prevent arginine conversion. Furthermore, as overexpression of the HB-Ub-tag reduces the availability of cellular d-biotin^21^, growth medium was supplemented with d-biotin (1 μM) to prevent saturation of in vivo biotinylation by excessive HB-Ub-tag expression. Plat-E retroviral packaging cells were maintained in DMEM supplemented with 10% (v/v) FBS, 100U ml^−1^ penicillin, 100 μM streptomycin, 1 μg ml^−1^ puromycin, 10 μg ml^−1^ blasticidin, and 1 mM glutamine.

### Constructs

Murine Grap2 (cat no. MR204666) and murine CIS (cat no. MR203328) in pCMV6-Entry were from Origene. Human SOCS3 CRISPR/Cas9 knockout (KO) and human SOCS3 HDR plasmids (cat. no. sc-400455) were from Santa Cruz Biotechnology. Expression constructs for Flag-tagged WT murine SOCS3 (hereafter termed pcDNA3/Flag-SOCS3), the truncation mutants ΔN20, ΔN36, ΔC40, and ΔC84, R71K-mutated SOCS3, and the ΔPEST mutant SOCS3 (generously provided by Dr Jeff Babon, Walter and Eliza Hall Institute of Medical Research, Australia) have all been described previously^30,31,33,71^. N-terminally GFP-tagged murine cavin-1 has been described previously^32^ while a cavin-1-mCherry expression construct was generously provided by Dr Ben Nichols (MRC Laboratory of Molecular Biology, Cambridge, UK).

Full-length murine WT SOCS3, ΔSH2 SOCS3 (amino acids 46–142), and SOCS box domain-only (amino acids 177–225) GFP fusion proteins were generated by PCR amplification and sub-cloning in-frame with the GFP open reading frame in pEGFP-N1 (Clontech). The following primers were used to generate PCR products using pcDNA3/Flag-SOCS3 as the template prior to digestion with the indicated restriction enzymes for sub-cloning:-

Forward primers: WT (GAA GAA GAA TTC GCC ACC *ATG* GTC ACC CAC AGC AAG), SOCS box only (GAA GAA GAA TTC GCC ACC *ATG* GTA CTG AGC CGA CCT CTC), SH2 domain only (GAA GAA GAA TTC GCC ACC *ATG* TTC TAC TGG AGC GCC GTG). *Eco*RI sites for sub-cloning underlined, initiating Met codon in italics. Reverse primers: WT and SOCS box only (TT CTC GGG ATC CGC AAG TGG AGC ATC ATA CTG ATC CAG G). ΔSH2 SOCS3 (TTC TCG GGA TCC GCT TCC GTG GGT GGC AAA G). *Bam*HI sites for sub-cloning underlined.

Myc-tagged WT murine cavin-1 and the truncation mutants N1 (amino acids 74–392), N2 (amino acids 168–392), N3 (amino acids 193–392), N4 (amino acids 250–392), C1 (amino acids 1–76), C2 (amino acids 1–167), C3 (amino acids 1–192), and C4 (amino acids 1–249) were generated by PCR amplification and sub-cloning in-frame with the C-terminal myc epitope (EQKLISEEDL) in pcDNA3.1/mycHis A(-) (Invitrogen). The following primers were used to generate PCR products using pEGFP-C1/cavin-1 as the template prior to digestion with the indicated restriction enzymes and sub-cloning:-

Forward primers: WT, C1, C3, C3 and C4 constructs (GGA GAA CCT CTA GAC GCC ACC *ATG* GAG GAT GTC ACG CTC), N1 (GGA GAA CCT CTA GAC GCC ACC *ATG* CAA GCC CAG CTG GAG), N2 (GGA GAA CCT CTA GAC GCC ACC *ATG* CTG AGC GTC AGC AAG TCG), N3 (GGA GAA CCT CTA GAC GCC ACC *ATG* CGG CCC GAG GAT GAC ACC), N4 (GGA GAA CCT CTA GAC GCC ACC *ATG* ACG CGT GAG AAC CTG GAG).* Xba*I sites for sub-cloning underlined, initiating Met codon in italics. Reverse primers: C1 (TTC TCG GAT CCA CTG GGC TTG GGT CAG CTG), C2 (TTC TCG GAT CCA TTT GGC CGG CAG CTT GAC), C3 (TTC TCG GAT CCA CTC GCC CTC GCC CAG CTC), C4 (TTC TCG GAT CCA GCG CAC CTT GGT CTT CTC). WT, N1, N2, N3, and N4 constructs (TTC TCG GAT CCA GTC GCT GTC GCT CTT GTC). *Bam*HI sites for sub-cloning underlined.

A mutated Grap2(SOCS3-PEST) in which residues 129–163 encompassing the SOCS3 PEST sequence were inserted between amino acids 149–150 within the Grap2 ORF in pCMV6-Entry was synthesised by GeneArt^TM^. All constructs were verified by sequencing to ensure the absence of additional unanticipated mutations.

### Retroviral delivery of a His_6_+biotin Ub (HB-Ub) transgene

Using Lipofectamine2000 (Invitrogen), Plat-E retroviral packaging cells in 10 cm dishes at approximately 80% confluence were transfected with a HB-Ub-expressing plasmid kindly donated by Professor Peter Kaiser (University of California at Irvine, USA)^22^. Following incubation in media without antibiotic selection, retrovirus-containing media were collected following two sequential incubation periods, one of 24 h at 37 °C and a second of 24 h at 32 °C.

### Retroviral-mediated generation of cell lines

MEFs in 10 cm dishes at approximately 40% confluence were transduced with 2 ml of retrovirus containing media in a final volume of 4 ml DMEM supplemented with 10% (v/v) FBS, 1 mM glutamine, and 10 μg ml^−1^ polybrene. After 12 h, the media were replaced with DMEM supplemented with 10% (v/v) FBS, 1 mM l-glutamine and 100U ml^−1^ penicillin, 100 μM streptomycin and 1 μg ml^−1^ puromycin to select for positive clones. Following dilution and re-plating, positive clones were expanded and HB-Ub-expressing clones identified by immunoblotting whole-cell extracts with a polyHis antibody.

### Tandem affinity purification

*SOCS3*^+/+^ and *SOCS3*^*−/−*^ MEFs were harvested in lysis buffer (8 M urea, 300 mM NaCl, 50 mM NaH_2_PO_4_, 0.5% (v/v) NP-40, pH 8.0) supplemented with 1 mM phenylmethylsulfonyl fluoride (PMSF). Following sonication (3 × 10 s pulses, with a 10 s rest phase, at 40% amplitude), supernatants were isolated by centrifugation at 21,000×*g* for 30 min at room temperature (RT) and equalised for protein content. Lysates from *SOCS3*^+/+^ and *SOCS3*^*−/−*^ MEFs were mixed in a 1:1 ratio before incubation with 30 μl of 50% (v/v) Ni^2+^-NTA-Sepharose beads per milligram of protein and rotated overnight at RT. Beads were isolated by centrifugation at 100×*g* for 1 min and then washed sequentially, once with 20 bead volumes of buffer A (8 M urea, 300 mM NaCl, 50 mM NaH_2_PO_4_, 0.5% (v/v) NP-40, pH 8.0) supplemented with 1 mM PMSF and 10 mM imidazole and twice with 20 bead volumes of buffer B (8 M urea, 300 mM NaCl, 50 mM NaH_2_PO_4_, 0.5% (v/v) NP-40, pH 6.3) supplemented with 10 mM imidazole and 1 mM PMSF. Beads were isolated by centrifugation at 100×*g* for 1 min and bound proteins eluted twice with 5 bead volumes of elution buffer (8 M urea, 200 mM NaCl, 50 mM NaH_2_PO_4_, 2% (w/v) SDS, 10 mM EDTA,100 mM Tris, 500 mM imidazole, pH 8.0) supplemented with 1 mM PMSF.

Eluate from the Ni affinity chromatography step was directly added to 10 μl of 50% (v/v) streptavidin-Sepharose beads per milligram of initial protein lysate and rotated overnight at RT. Beads isolated by centrifugation at 100×*g* for 1 min at RT were washed sequentially, twice with 25 bead volumes of buffer C (8 M urea, 200 mM NaCl, 2% (w/v) SDS, 100 mM Tris, pH 8.0) and twice with 25 bead volumes of buffer D (8 M urea, 1.2 M NaCl, 0.2% (w/v) SDS, 100 mM Tris, 10% (v/v) ethanol, 10% (v/v) isopropanol, pH 8.0). Bound proteins were eluted with one bead volume of aqueous biotin (50 mM) at 95 °C for 5 min. Following isolation by centrifugation at 100×*g* for 1 min at RT, eluate was concentrated using Amicon 10K Ultra-2 Centrifugal Filter Devices (Millipore) as per the manufacturer’s instructions.

### In-gel trypsin digestion

Sodium dodecyl sulphate–polyacrylamide gel electrophoresis (SDS-PAGE)-fractionated TAP eluate was stained with InstantBlue (Expedion) prior to manual sectioning into several manageable gel slices. Individual gel slices were washed sequentially with 500 μl, 100 mM ammonium bicarbonate and then 500 μl, 50% (v/v) acetonitrile/ammonium bicarbonate (100 mM) for 30 min with shaking. The samples were reduced with the addition of 150 μl 100 mM ammonium bicarbonate and 10 μl 45 mM dithiothreitol for 30 min at 60 °C. Samples were cooled to RT before alkylation using 10 μl 100 mM iodoacetamide in the dark for 30 min at RT. Gel pieces were then washed in 500 μl 50% (v/v) acetonitrile/ammonium bicarbonate (100 mM) for 1 h with shaking at RT. Following treatment with 50 μl acetonitrile for 10 min, the solvent was discarded and the gel pieces dried using a vacuum centrifuge for 1 h. Gel slices were fully rehydrated in trypsin suspended in 1 ml 25 mM ammonium bicarbonate and incubated overnight at 37 °C after which the supernatant was transferred to a fresh 96-well plate without disturbing the gel pieces. Residual digested protein was extracted by using 20 μl 5% (v/v) formic acid for 20 min at RT with shaking followed by the addition of 40 μl acetonitrile for a further 20 min with shaking at RT. Pooled extracts were dried using a SpeedVac centrifugal evaporator before resuspension in 10 μl dH20 prior to mass spectrometry.

### Liquid chromatography and mass spectrometry

Samples were analysed on a Dionex Ultimate 3000 RSLS Nano flow system (Dionex). The samples (5 μl) were loaded onto a Dionex 100 μm × 2 cm 5 μm C18 Nano trap column at a flow rate of 5 μl min^−1^ by the Ultimate 3000 RS autosampler (Dionex). The composition of the loading solution was 0.1% formic acid and acetonitrile (98:2). Once loaded onto the column, the sample was then washed off into an Acclaim PepMap 75 μm × 15 cm, 2 μm 100 Å C18 Nano column at a flow rate of 0.3 μm min^−1^. The trap and nano flow column were maintained at 35 °C in an UltiMate 3000 Rapid Separation LC system (Thermo Fisher). The samples were eluted with a gradient of solvent A: 0.1% formic acid (solvent A) versus acetonitrile (solvent B) starting at 1% B rising to 15% then to 45% B over 50 and then 90 min. The column was washed using 90% B before being equilibrated prior to the next sample being loaded.

Column eluate was directed to a Proxeon Nano spray electrospray ionisation (ESI) source (Thermo Fisher) operating in positive ion mode and then into an Orbitrap Velos FTMS. The ionisation voltage was 2.5 kV and the capillary temperature was 230 °C. The mass spectrometer was operated in tandem mass spectrometry (MS/MS) mode scanning from 300 to 2000 amu. The top 20 multiply charged ions were selected from each full scan for MS/MS analysis, the fragmentation method was CID at 35% collision energy. The ions were selected for MS^2^ using a data-dependent method with a repeat count of 1 s and repeat and exclusion time of 15 s. Precursor ions with a charge state of 1 were rejected. The resolution of ions in the first stage (MS^1^) was 60,000 and 7500 for the second stage (CID MS^2^). Data were acquired using Xcalibur v.2.1 (Thermo Fisher).

### Analysis of LC-MS/MS

Post-LC-MS/MS analysis was performed using MaxQuant v.1.1.1.36^72^ and searched with Andromeda search engine^73^ against the IPI mouse.v3.80 Fasta formatted database (release February 2011). Phosphorylation (S, T, Y), ubiquitination (GlyGly), and oxidation (Met) were set as variable modifications, whereas carbamidomethylation (Cys) was set as fixed modification. The peptides used for protein quantification were set to unique and razor and minimum ratio count set to 1. Requantify was set to “TRUE” for deep searching of paired SILAC peaks. “Labelled amino acid filtering” was set to “FALSE” to improve analysis using R6K6 SILAC labelling. All other options were set to default.

### CRISPR/Cas9 generation of SOCS3-null AS-M.5 EC lines

Using SuperFect transfection reagent (Qiagen), AS-M.5 cells in 6 cm dishes at approximately 80% confluence were co-transfected with human SOCS3 CRISPR/Cas9 KO and human SOCS3 HDR plasmids. Following dilution and re-plating, positive clones were isolated by selection in medium supplemented with puromycin (2 μg ml^−1^). SOCS3-null clones were identified by immunoblotting whole-cell extracts with SOCS3 antibody following cellular treatment with Fsk (50 μM, 5 h) to induce SOCS3 gene expression^26,45^.

### Antibodies

The following antibodies were obtained from the indicated suppliers: anti-Flag M2 antibody (Sigma F3165, 1 in 1000), anti-HA HA-7 antibody (Sigma H9658, 1 in 1000), PTRF/cavin-1 (Abcam ab48824, 1 in 1000), caveolin-1 (BD Biosciences 610059, 1 in 1000) and anti-phosphotyrosine monoclonal antibody 4G10 (Millipore 05–321, 1 in 1000), GAPDH (Abcam, ab8245, 1 in 20,000), anti-myc 9E10 (ascites generated by ProSci, 1 in 2000), anti-α-tubulin 12G10 (DSHB 12G10, 1 in 10,000), SOCS3 (M20; Santa Cruz sc-7009, 1 in 500), STAT3 (EPR787Y: Abcam ab68153, 1 in 1000), phospho-STAT3 (Tyr705) (3E2: Cell Signaling 9138L, 1 in 1000), phospho-STAT3 (Ser727) (6E4: Cell Signaling 9136, 1 in 1000), JAK1 (BD Transduction Laboratories 610232, 1 in 1000), JAK2 (D2E12: Cell Signaling 3230, 1 in 1000), and flotillin-1 (BD Transduction Laboratories 610821, 1 in 500). Sheep polyclonal anti-GFP serum was generously provided by Professor Graeme Milligan (University of Glasgow, UK) and was used in a 1 in 2000 dilution.

### Immunoblotting

Cell lysates were prepared as described previously^74^. Cells were washed twice with ice-cold phosphate-buffered saline (PBS) and lysed by scraping into lysis buffer (50 mM HEPES pH 7.4, 150 mM sodium chloride, 1% (v/v) Triton X-100, 0.5% (v/v) sodium deoxycholate, 0.1% (w/v) SDS, 10 mM sodium fluoride, 5 mM EDTA, 10 mM sodium phosphate, 0.1 mM PMSF, 10 μg ml^−1^ benzamidine, 10 μg ml^−1^ soybean trypsin inhibitor, 2% (w/v) EDTA-free complete protease inhibitor cocktail (Sigma)). After 30 min on ice, lysates were vortexed and cleared by centrifugation. Equivalent amounts of protein, as determined by bicinchoninic acid protein assay, were resolved by SDS-PAGE, transferred to a nitrocellulose membrane, and analysed by immunoblotting as previously described^26,74,75^. Uncropped immunoblots used to generate Figs. 1b and 5b are shown in Supplementary Figure 7.

### RNA analysis

Total RNA extraction from MEFs grown in 60 mm dishes was carried out using a miRNeasy Mini Kit (Qiagen) according to the manufacturer’s instructions. The cDNA was generated from 1 μg total RNA using 200 U SuperScript™ II Reverse Transcriptase (Invitrogen) following the manufacturer’s instructions with 100 ng random hexamers, 2.5 mM of each dNTP and 40 U RNaseOUT (Invitrogen) in a final volume of 20 μl. Real-time quantitative PCRs were performed on a MX3000 system (Stratagene) using Power SYBR^®^ Green PCR Master Mix (Applied Biosystems) in a final volume of 10 μl containing 1 μl cDNA, 0.5 mM of each primer, and 1x Power SYBR^®^ Green PCR Master Mix. The murine cavin-1 primers used were GCAAGGTCAGCGTCAAC (forward primer) and CCGGCAGCTTGACTTCA (reverse primer). GAPDH primers used for normalisation were GGCTGGCATTGCTCTCAA (forward primer) and GCTGTAGCCGTATTCATTGTC (reverse primer). Primers were purchased from Dharmacon.

### Co-immunoprecipitation and peptide pull-down assays

For co-IP assays, either transfected cells or WT and SOCS3-null AS-M.5 cells were harvested in ice-cold PBS, pelleted by centrifugation at 1000×*g* for 5 min at 4 °C, and lysed in co-IP buffer (50 mM HEPES, pH 7.4, 120 mM NaCl, 5 mM EDTA, 10% (v/v) glycerol, 1% (v/v) Triton X-100, 5 mM NaF, 1 mM sodium orthovanadate, 10 μg ml^−1^ benzamidine, 0.1 mM PMSF, 10 μg ml^−1^ soybean trypsin inhibitor, 2% (w/v) EDTA-free complete protease inhibitor cocktail). Following solubilisation by incubation for 1 h at 4 °C with rotation, lysates were centrifuged at 21,000×*g* for 15 min at 4 °C and the supernatant equalised for protein content and volume. Non-specifically binding proteins were removed from soluble fractions by a 1 h pre-clearing step using 40 μl of 50% (v/v) slurry of protein G-Sepharose 4B FF beads (Sigma) re-suspended in 100 μl 2% (w/v) IgG-free bovine serum albumin (BSA). Following sedimentation of protein G-Sepharose beads by brief centrifugation, pre-cleared lysates were incubated overnight at 4 °C with rotation with either 40 μl fresh protein G-Sepharose beads pre-equilibrated with 2% (w/v) IgG-free BSA and anti-cavin-1 antibody or 40 μl pre-conjugated anti-Flag M2-agarose beads (Sigma). Immune complexes were then isolated by brief centrifugation and washed three times with 1 ml of co-IP buffer. Following removal of the final wash, protein complexes were eluted for analysis by SDS-PAGE by the addition of 40 μl of electrophoresis sample buffer containing 12% (w/v) SDS and incubation for 30 min at 65 ^o^C followed by a further 5 min at 95 °C.

For peptide pull-down assays, protein-equalised soluble extracts from transfected HEK293 cells were incubated with 100 nM N-terminally biotinylated peptides (Severn Biotech, UK) and streptavidin-agarose prior to isolation of complexes by brief centrifugation and washing as described above. The peptides used had the following amino acid sequences: Tyr759 (Y), biotin-TSSTVQYSTVVHSG; and PTyr759 (pY), biotin-TSSTVQpYSTVVHSG). Samples were then eluted for analysis by SDS-PAGE and immunoblotting as described above.

### Peptide array overlays

Arrays were produced by automatic SPOT synthesis and synthesised on continuous cellulose membrane supports on Whatman 50 cellulose membranes using Fmoc-chemistry with the AutoSpot-Robot ASS 222 (Intavis Bioanalytical Instruments AG) as we have previously described^76^. Following blocking of non-specific protein binding sites by incubation in tris-buffered saline with Tween-20 (TBST; 50 mM Tris pH 7.5, 150 mM NaCl, 0.1% (v/v) Tween 20) containing 5% (w/v) BSA, membranes were overlaid with 10 μg ml^−1^ purified recombinant Trx-polyHis-tagged SOCS3 (Sino Biological Inc.) diluted in TBST-5% (w/v) BSA. After washing in TBST, bound SOCS3 was detected by probing overlays with anti-SOCS3 antibody followed by IRDye-conjugated secondary antibody prior to visualisation using a LI-COR Odyssey Sa imaging system. As a negative control, identical arrays were identically treated in parallel minus recombinant SOCS3.

### Subcellular fractionation

Confluent 10 cm dishes of WT, *cavin-1*^*−/−*^, and *SOCS3*^*−/−*^ MEFs were used to prepare subcellular fractions using a Subcellular Protein Fractionation Kit (Thermo Scientific) in accordance with the the manufacturer’s instructions.

### Confocal microscopy

For analysis of endogenous cavin-1 and transfected SOCS3-GFP, WT, and *cavin-1*^*−/−*^ MEFs in 10 cm dishes were transiently transfected with or without SOCS3-GFP expression constructs. The following day, cells were split onto glass coverslips and left for a further 24 h. Cells were then washed with PBS and fixed with 3% (w/v) paraformaldehyde (PFA) in PBS for 25 min. After washing with PBS and quenching residual PFA with 20 mM glycine in PBS, cells were permeabilised with 0.1% (v/v) Triton X-100 and non-specific binding sites blocked by a 30 min of incubation at RT in PBS containing 3% (w/v) BSA and 10% (v/v) donkey serum. Cells were then incubated with rabbit anti-cavin-1 antibody (Abcam ab48824, 1 in 100 dilution) for 90 min at RT. Cells were washed with PBS containing 0.1% (v/v) Triton X-100, 1% (w/v) BSA, and 10% (v/v) donkey serum prior to incubation with Alexa Fluor 594-conjugated donkey anti-rabbit IgG (Life Technologies A21207, 1 in 200 dilution) for 1 h at RT. Finally, the cells were washed with PBS, mounted in ProLong*®* Gold anti-fade reagent containing nuclear stain 4’,6-diamidino-2-phenylindole (DAPI), and visualised using a LSM510 laser scanning confocal imaging system (Carl Zeiss). Images were analysed by Metamorph software to generate Pearson’s correlation coefficients.

For experiments involving co-expression of SOCS3-GFP and cavin-1-mCherry in *cavin-1*^*−/−*^ MEFs, cells at 80–90% confluence on 6 cm dishes were transfected with 1 µg of each construct using PolyFect transfection reagent as per the manufacturer’s instructions. The following day, cells were seeded into ×16 Lab-Tek chamber slides (Fisher Scientific) at a density of 5 × 10^4^ cells per chamber and cultured for a further 24 h. Cells were then washed twice with Hanks' balanced salt solution with Ca^2+^/Mg^2+^ and 0.2% (w/v) sucrose to preserve morphology before fixing by incubation with 4% (w/v) PFA at RT for 20 min in the dark. After removal of fixative and two washes with PBS, nuclei were stained with 10 µg ml^−1^ Hoechst 33342 (Life Technologies) prior to imaging using a VivaTome spinning disk confocal microscope (Carl Zeiss). Images were analysed using Fiji ImageJ and the Coloc 2 plugin.

### Transmission electron microscopy

WT and SOCS3-null AS-M.5 cells were seeded at a density of 1 × 10^6^ cells per ml into 6-well plates and onto Thermanox coverslips (13 mm diameter) for culturing to confluency. The cells were then fixed in 1.5% (w/v) glutaraldehyde in 0.1 M sodium cacodylate buffer at 4 °C for 1 h. Following three washes in 0.1 M sodium cacodylate buffer in 2% (w/v) sucrose, the cells were incubated with 1% (w/v) osmium tetroxide/0.1 M sodium cacodylate for 1 h, washed three times in distilled water, and incubated in 0.5% (w/v) uranyl acetate in the dark for 1 h. Following two rinses in distilled water, cells were dehydrated in stepwise alcohol increments (30–100% (v/v)) and incubated overnight in a 1:1 mix of propylene oxide/TAAB araldite Epon 812 resin. The propylene oxide was then allowed to evaporate to leave pure resin, which was changed twice before the sample was embedded in fresh resin which was allowed to polymerise at 60 °C for 48 h. Ultrathin sections were cut using a Leica Ultracut UCT and a Diatome diamond knife, contrast stained with aqueous 2% (w/v) methanolic uranyl acetate and Reynolds lead citrate, and viewed using a LEO 912AB TEM (Carl Zeiss) at an accelerating voltage of 120 kV. TIF images were captured using an Olympus Soft Imaging System and image contrast modified using Adobe Photoshop CS.

### Statistics

Statistical significance was assessed either by one-way analysis of variance or unpaired *t*-tests with an *α* probability of 0.05. At least three separate experiments were used for analysis.

### Data availability

All the data that support the findings of this study are available from the corresponding author upon reasonable request.

## Electronic supplementary material

Supplementary Information
