# Supplementary material for: Interaction of suppressor of cytokine signalling 3 with cavin-1 links SOCS3 function and cavin-1 stability
Source: Nat Commun. 2018 Jan 12;9:168. doi: 10.1038/s41467-017-02585-y (PMC5766592; doi:10.1038/s41467-017-02585-y)
Supplement: Supplementary file 1 — Supplementary Information [file 41467_2017_2585_MOESM1_ESM.pdf]

SUPPLEMENTARY INFORMATION

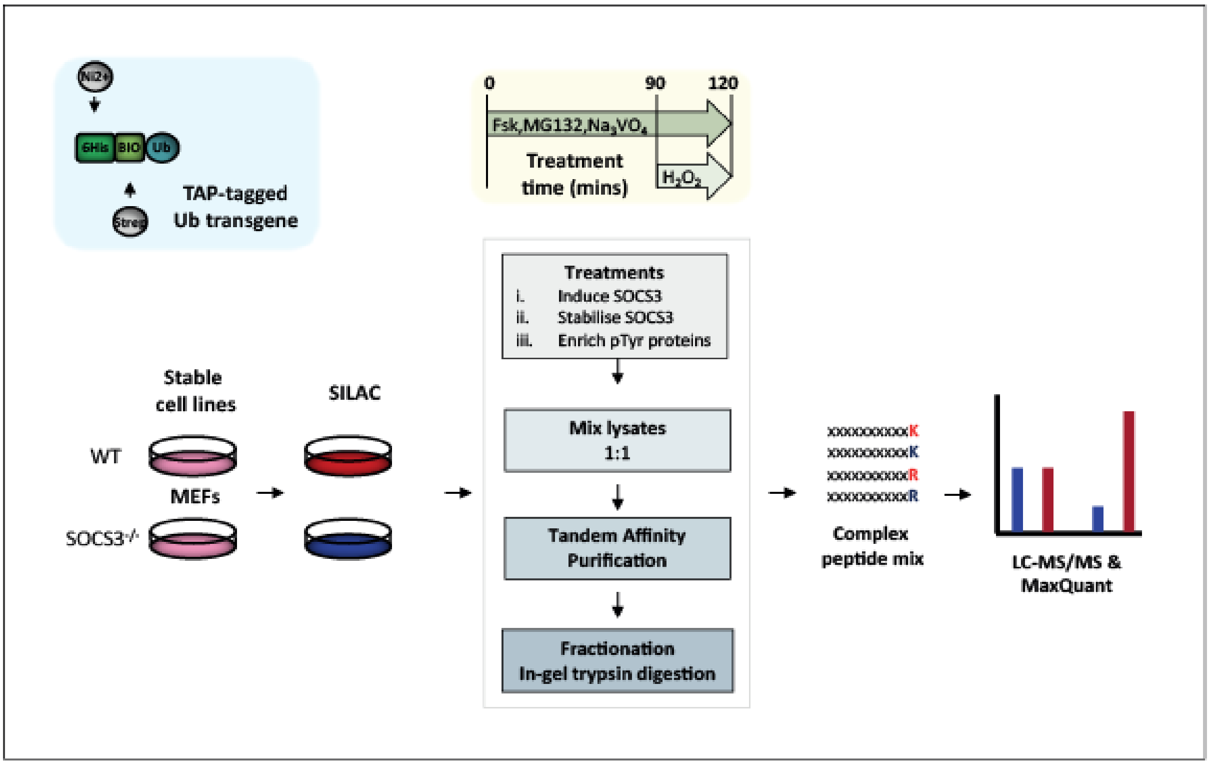

Supplementary Figure 1:

Differentially SILAC-labelled, tandem affinity purified ubiquitinomes isolated from WT MEFs and *SOCS3*$^{-/-}$ MEFs each expressing tagged ubiquitin transgenes (blue box) were compared following induction SOCS3 by treatment with forskolin (50 µM) for 2 hrs. Proteasomal degradation of SOCS3 and potential ubiquitinated substrates was inhibited by co-incubation with proteasome inhibitor MG132 (6 µM) while Tyr phosphorylation status was preserved through inhibition of protein Tyr phosphatases by incubation with sodium orthovanadate (Na_3VO_4: 1 mM) for 1.5 hrs then hydrogen peroxide (H_2O_2: 0.2 mM) for a further 30 mins prior to harvesting. Tandem-affinity-purified proteins were digested with trypsin and peptides processed by sequential liquid chromatography and mass spectrometry.

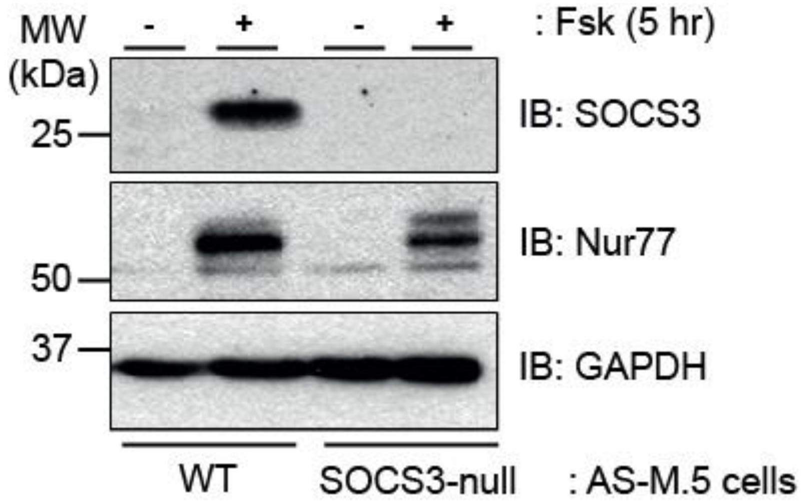

Supplementary Figure 2: Detergent-soluble whole cell lysates from WT and SOCS3-null AS-M.5 human angiosarcoma-derived ECs treated with either vehicle or 50 μM Fsk for 5 hr were equalised for protein content for SDS-PAGE for immunoblotting with the indicated antibodies. Data shown are representative of multiple experiments.

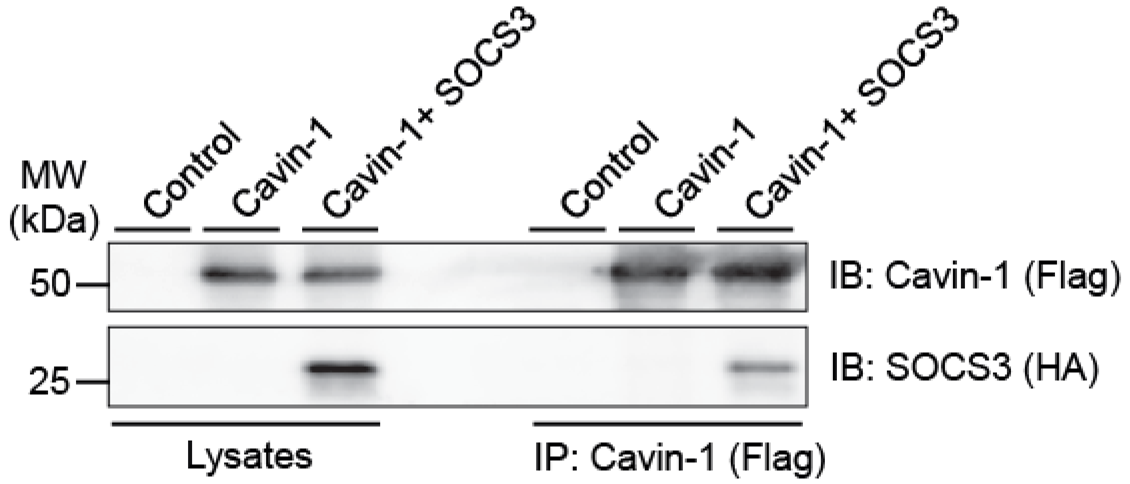

Supplementary Figure 3: Protein-equalised soluble cell extracts from HEK293 cells transfected with expression constructs encoding HA-tagged SOCS3 and Flag-tagged cavin-1 as indicated were processed by immunoprecipitation (IP) with anti-Flag M2-agarose beads prior to SDS-PAGE and immunoblotting with the indicated antibodies. Whole cell lysates from the samples used in the IP were also analysed in parallel.

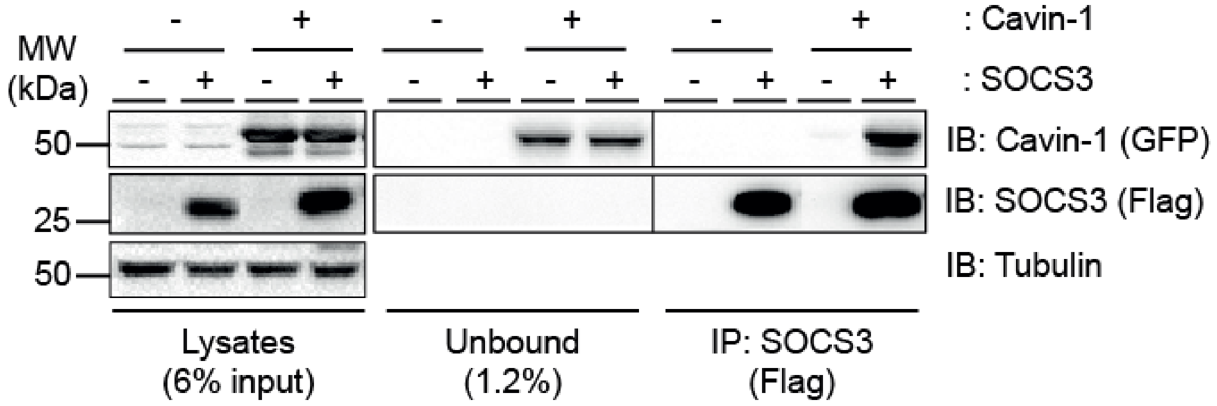

Supplementary Figure 4: Protein-equalised soluble cell extracts from HEK293 cells transfected with expression constructs encoding Flag-SOCS3 and GFP-tagged cavin-1 as indicated were processed by immunoprecipitation (IP) with anti-Flag M2-agarose beads prior to SDS-PAGE and immunoblotting with the indicated antibodies. Fractions of the whole cell lysates from the samples and unbound supernatants from the IP were also fractionated by SDS-PAGE for immunoblotting in parallel. Data shown are representative of three experiments.

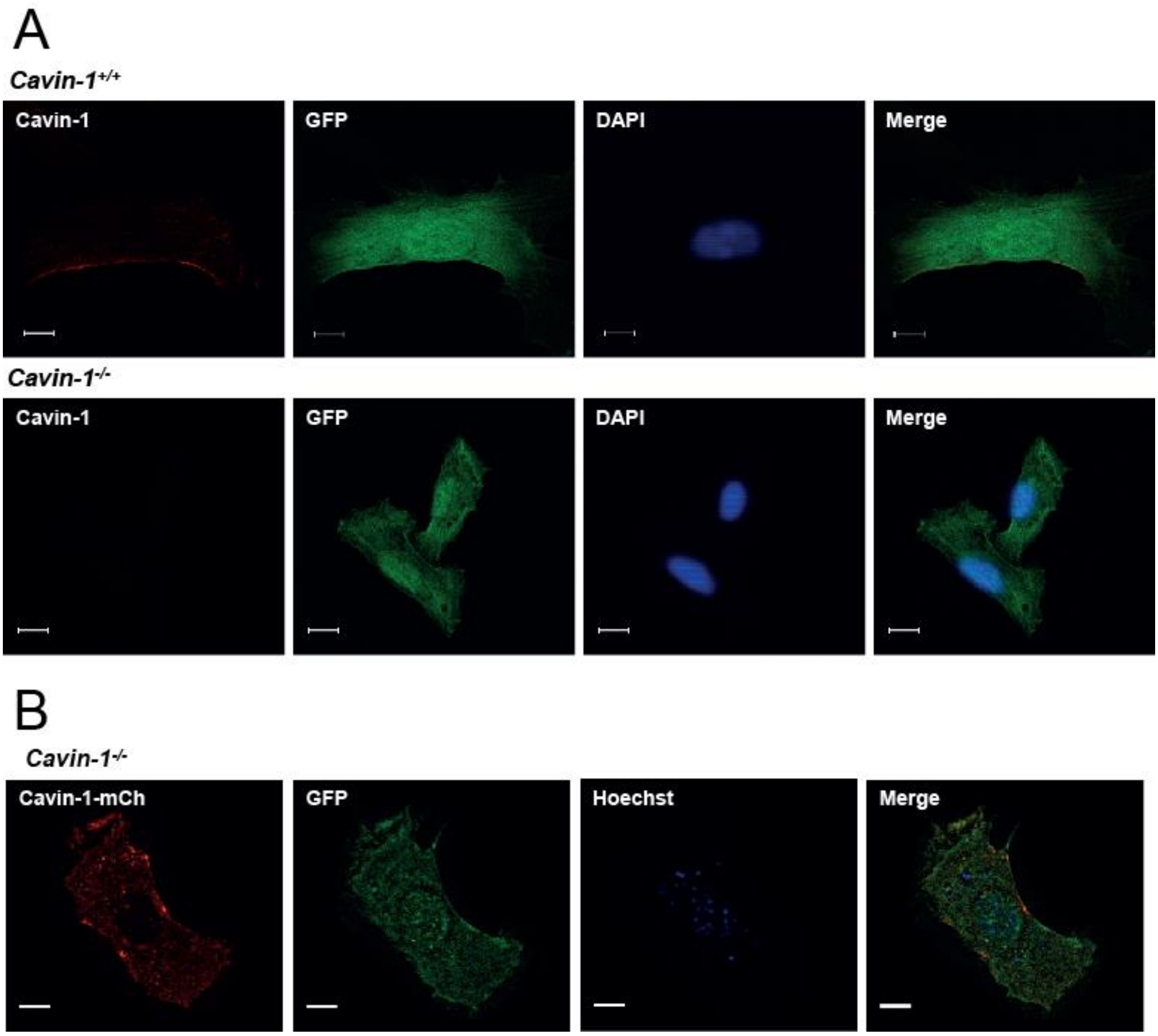

Supplementary Figure 5:

(A) WT (*cavin-1*$^{+/+}$) and *cavin-1*$^{-/-}$ MEFs transiently expressing GFP (green) were stained with DAPI prior to being fixed, solubilised and stained with anti-cavin-1 antibody (red) before mounting for imaging by confocal microscopy. Scale bar = 10 μm.

(B) *Cavin-1*$^{-/-}$ MEFs transiently co-expressing GFP (green) and cavin-1-mCh (red) were stained with Hoechst 33342 prior to being fixed for imaging by confocal microscopy. Scale bar = 10 μm.

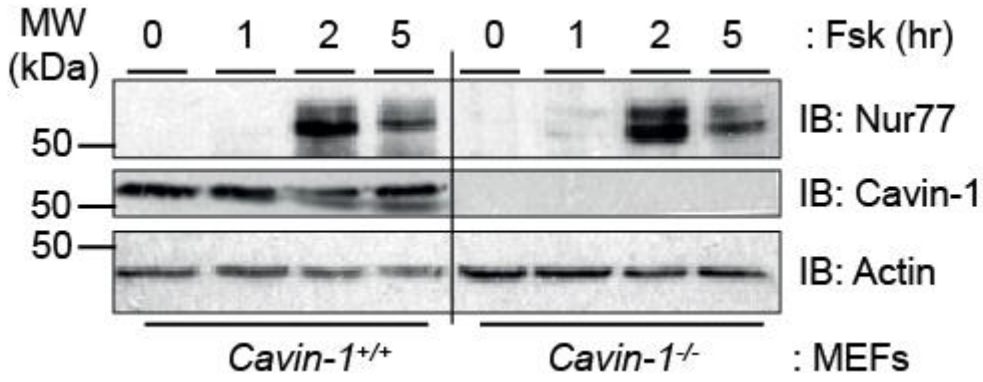

Supplementary Figure 6: Detergent-soluble whole cell lysates from *cavin-1*$^{+/+}$ and *cavin-1*$^{-/-}$ MEFs treated with either vehicle or 50 μM Fsk for the indicated times were equalised for protein content for SDS-PAGE for immunoblotting with the indicated antibodies. Data shown are representative of three experiments.

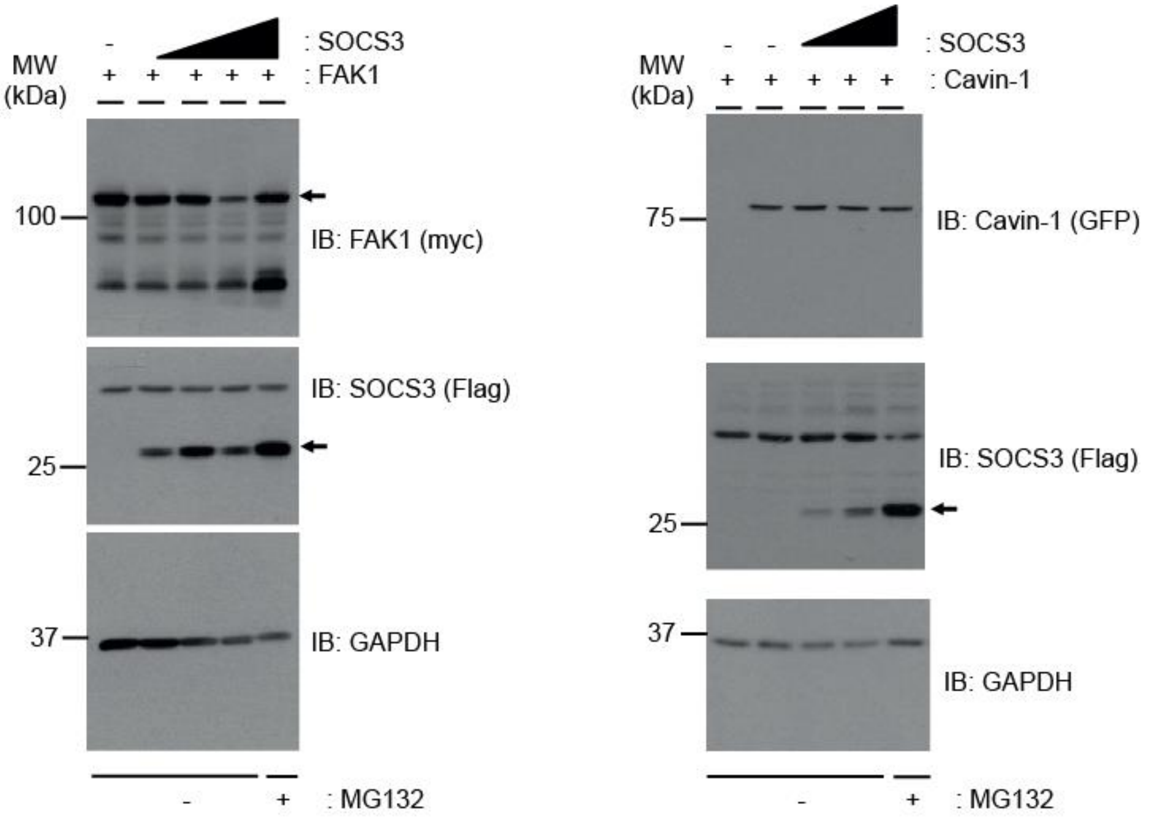
: SOCS3
MW (kDa)
: FAK1
100
IB: FAK1 (myc)
IB: SOCS3 (Flag)
25
37
IB: GAPDH
: MG132
: SOCS3
: Cavin-1
75
IB: Cavin-1 (GFP)
IB: SOCS3 (Flag)
25
37
IB: GAPDH
: MG132

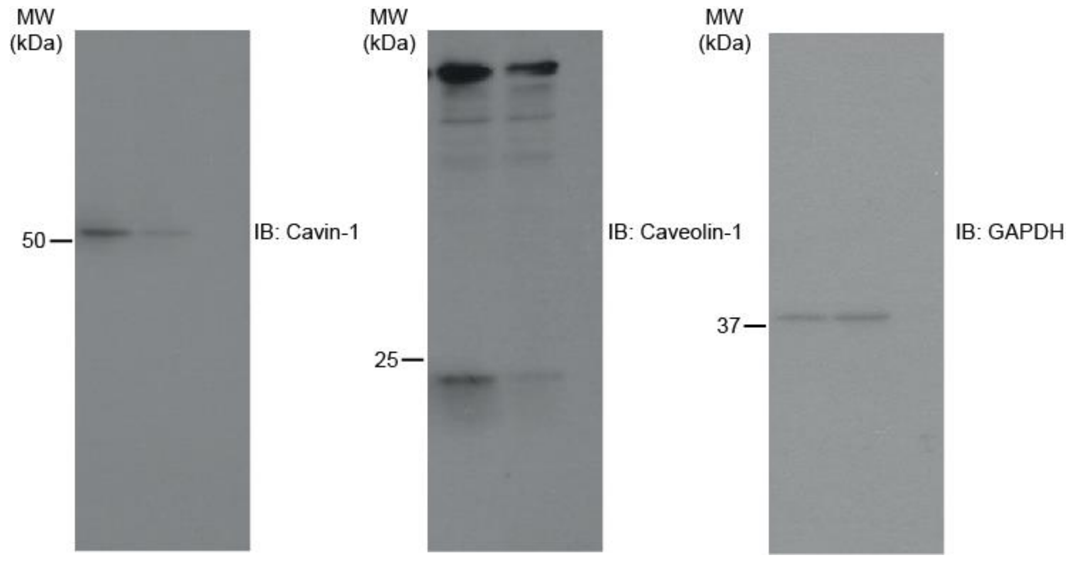
MW (kDa)
50
IB: Cavin-1
MW (kDa)
25
IB: Caveolin-1
MW (kDa)
37
IB: GAPDH

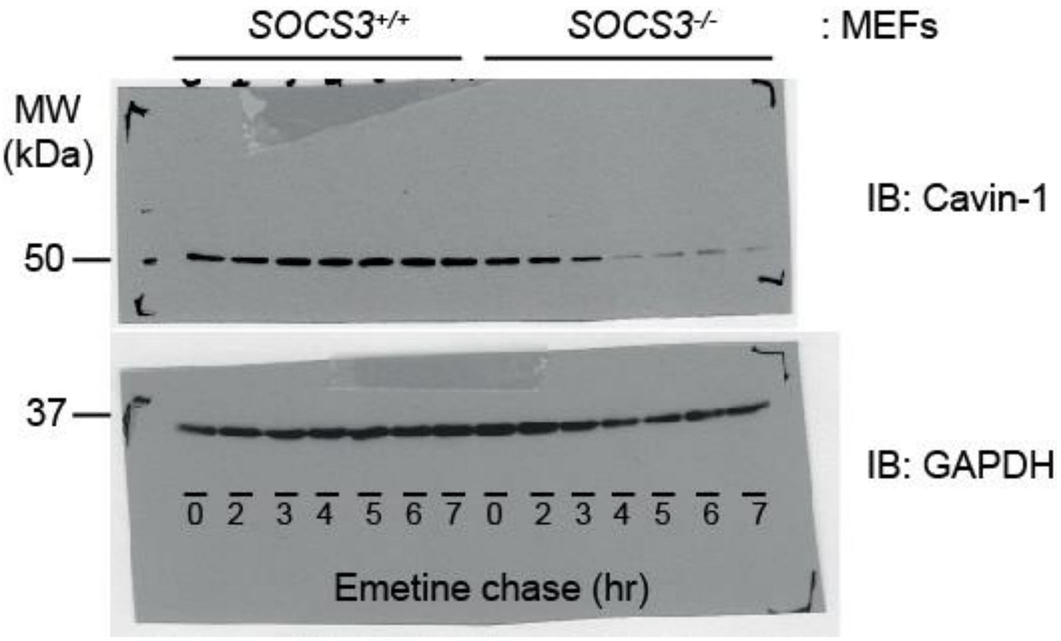

SOCS3+/+
SOCS3-/-
: MEFs
MW (kDa)
50
37
IB: Cavin-1
IB: GAPDH
0 2 3 4 5 6 7 0 2 3 4 5 6 7
Emetine chase (hr)

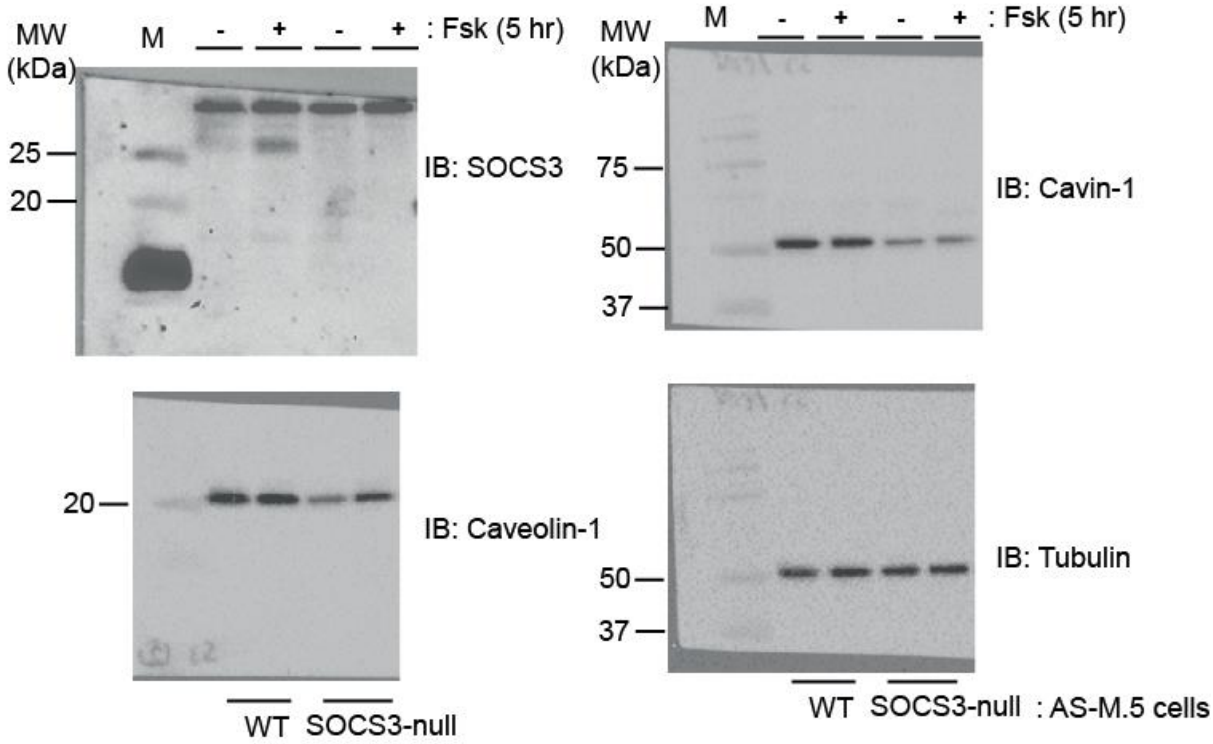

MW (kDa)
M
- + - + : Fsk (5 hr)
25
20
IB: SOCS3
IB: Caveolin-1
WT
SOCS3-null
75
50
37
IB: Cavin-1
IB: Tubulin
: AS-M.5 cells

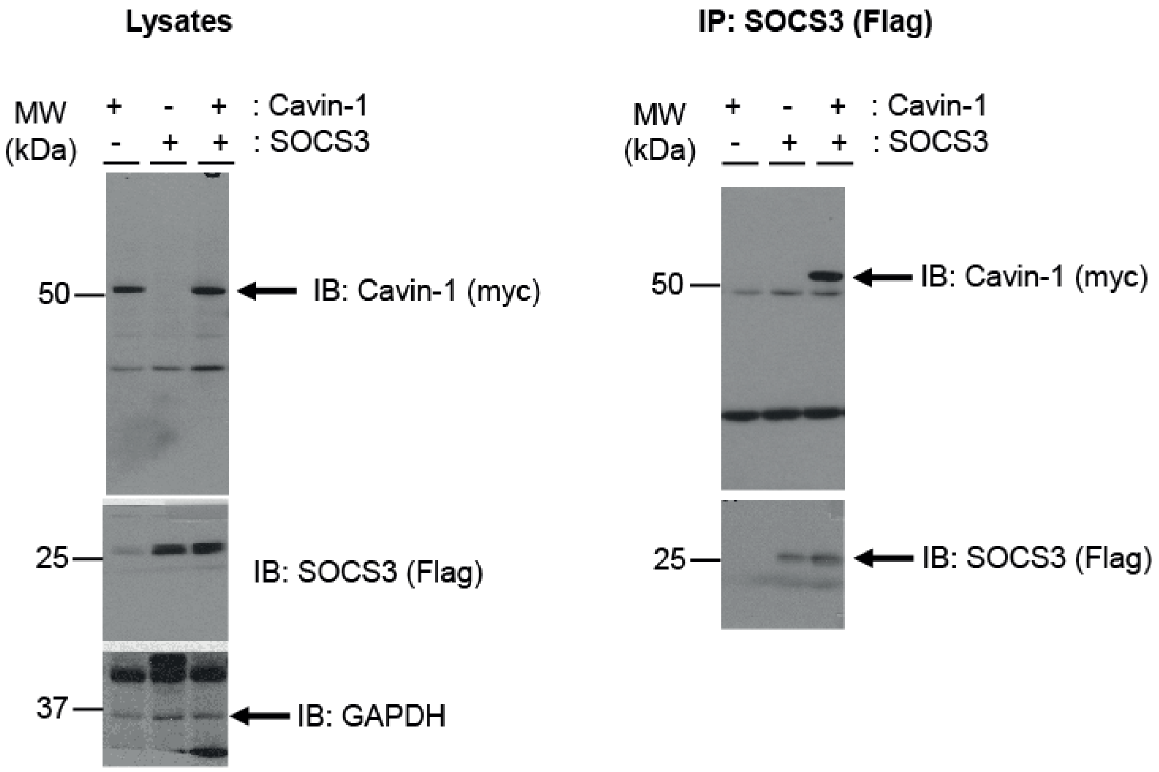
Lysates
IP: SOCS3 (Flag)
MW (kDa)
+ - + : Cavin-1
- + + : SOCS3
50
IB: Cavin-1 (myc)
25
IB: SOCS3 (Flag)
37
IB: GAPDH

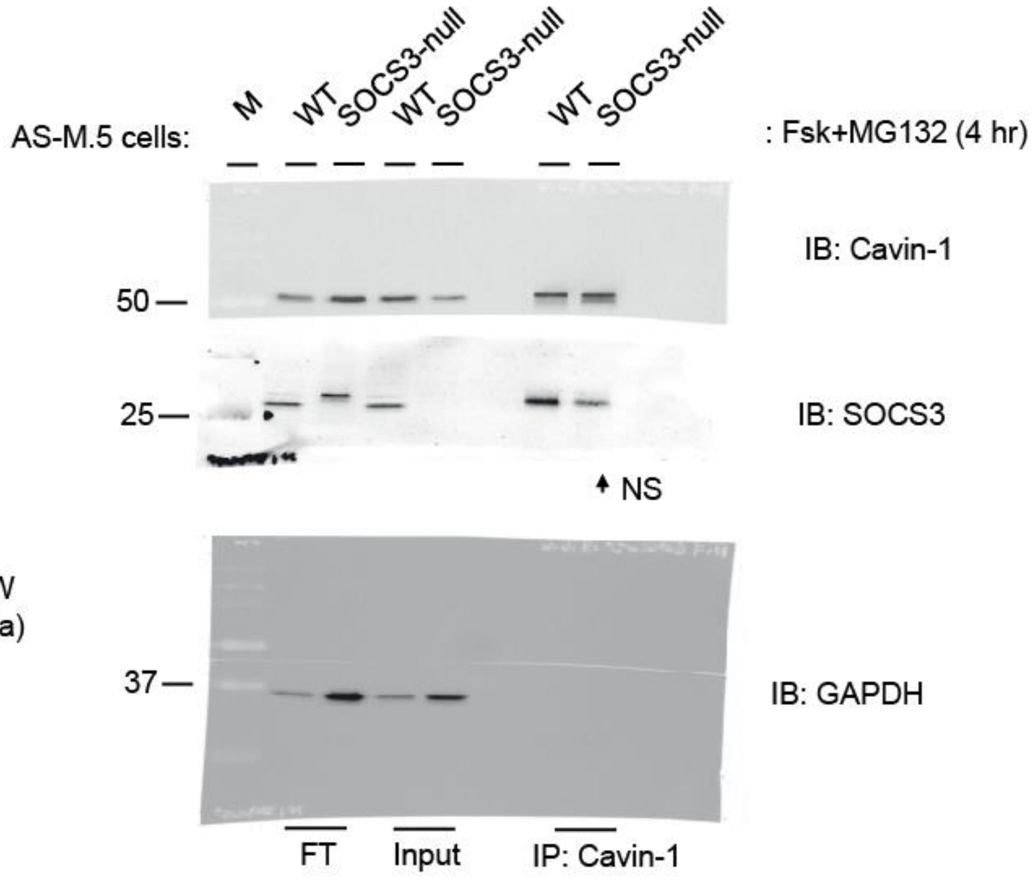
AS-M.5 cells:
M
WT
SOCS3-null
WT
SOCS3-null
WT
SOCS3-null
: Fsk+MG132 (4 hr)
IB: Cavin-1
50
25
IB: SOCS3
NS
MW (kDa)
37
IB: GAPDH
FT
Input
IP: Cavin-1

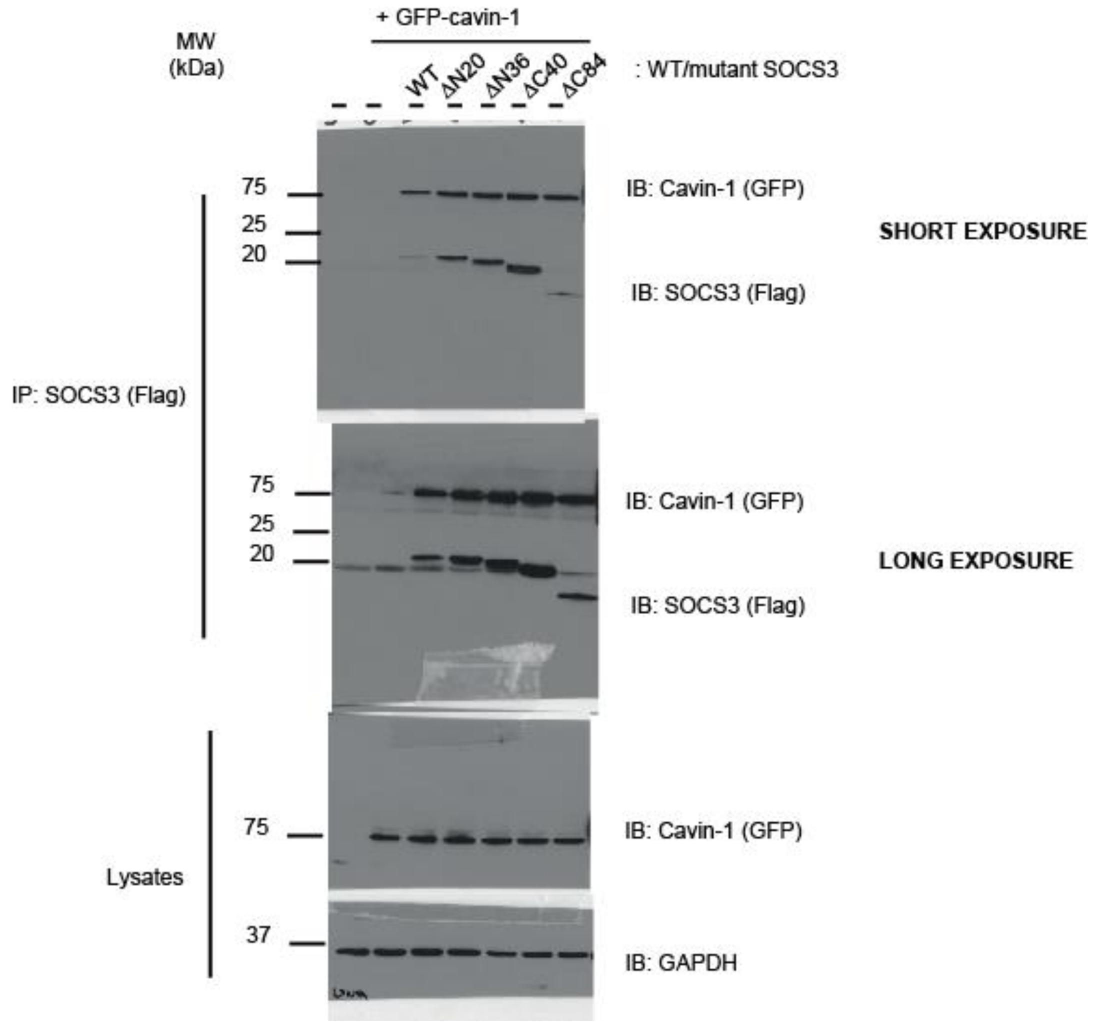

+ GFP-cavin-1
MW (kDa)
WT ΔN20 ΔN36 ΔC40 ΔC84
: WT/mutant SOCS3
75
25
20
IB: Cavin-1 (GFP)
IB: SOCS3 (Flag)
SHORT EXPOSURE
IP: SOCS3 (Flag)
LONG EXPOSURE
Lysates
37
IB: GAPDH

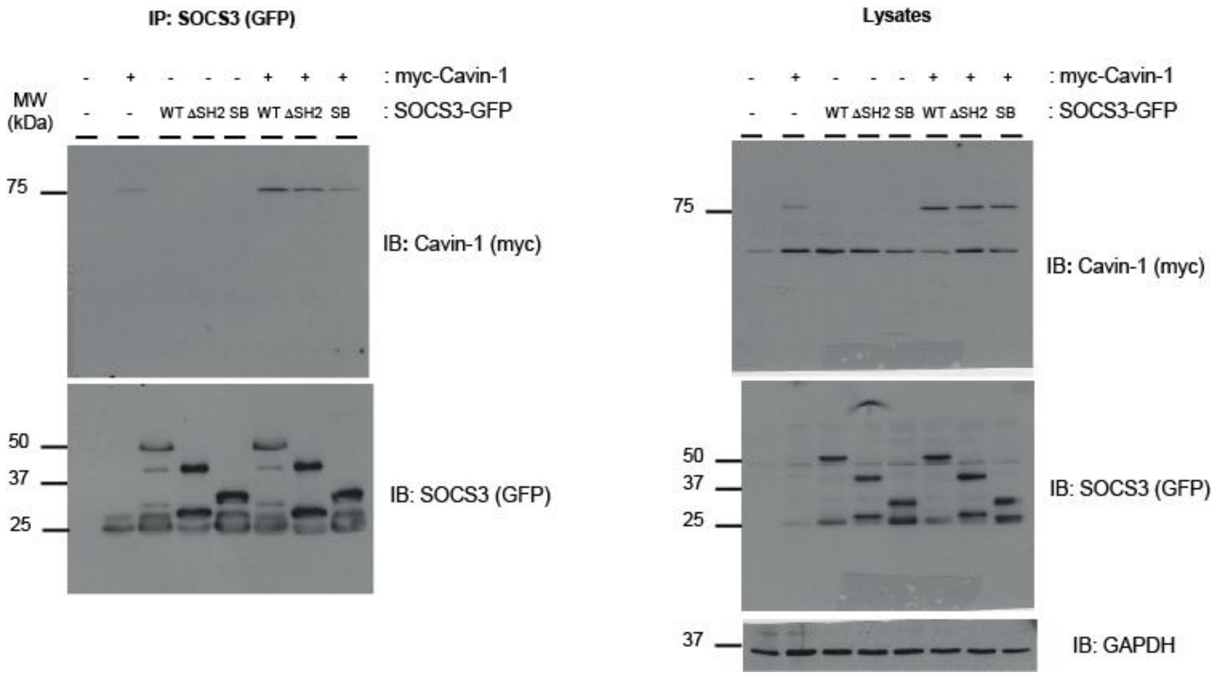

IP: SOCS3 (GFP)
- + - - - + + + : myc-Cavin-1
MW (kDa)
- - WT ΔSH2 SB WT ΔSH2 SB : SOCS3-GFP
75
IB: Cavin-1 (myc)
50
37
25
IB: SOCS3 (GFP)
Lysates
- + - - - + + + : myc-Cavin-1
- - WT ΔSH2 SB WT ΔSH2 SB : SOCS3-GFP
75
IB: Cavin-1 (myc)
50
37
25
IB: SOCS3 (GFP)
37
IB: GAPDH

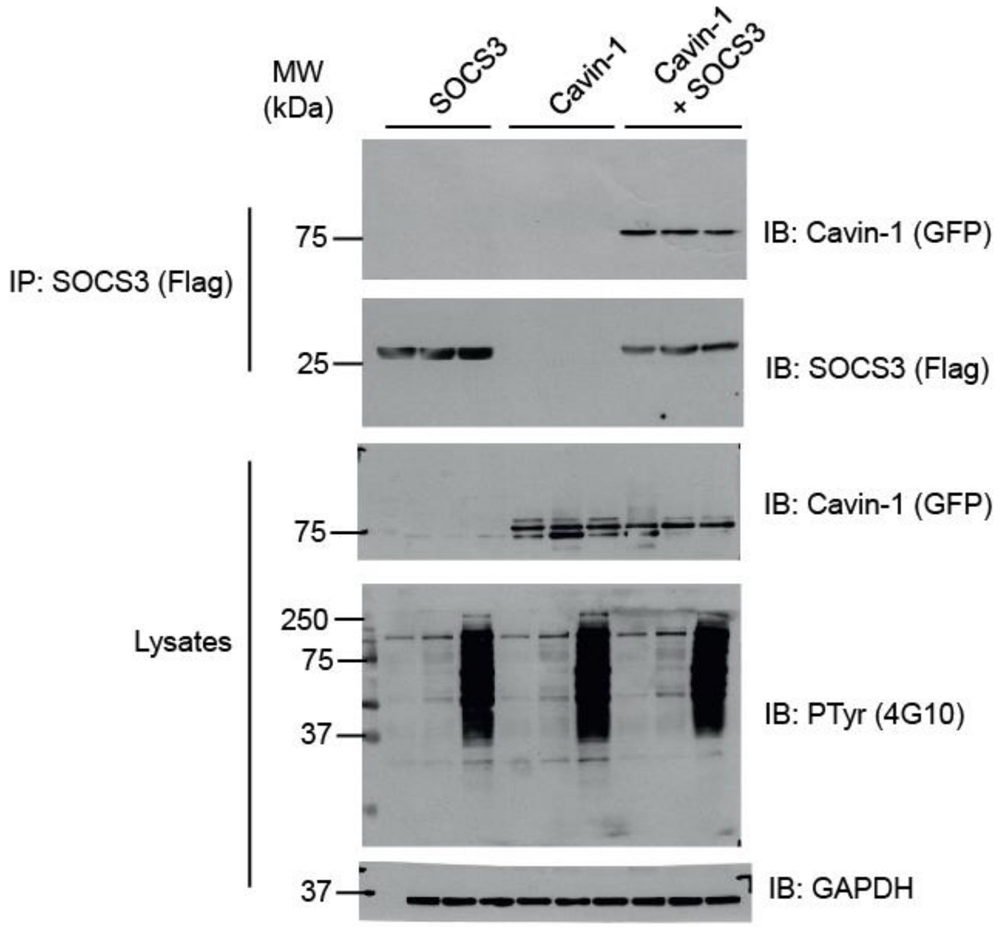
MW (kDa)
SOCS3
Cavin-1
Cavin-1 + SOCS3
IP: SOCS3 (Flag)
75
IB: Cavin-1 (GFP)
25
IB: SOCS3 (Flag)
Lysates
75
IB: Cavin-1 (GFP)
250
75
37
IB: PTyr (4G10)
37
IB: GAPDH

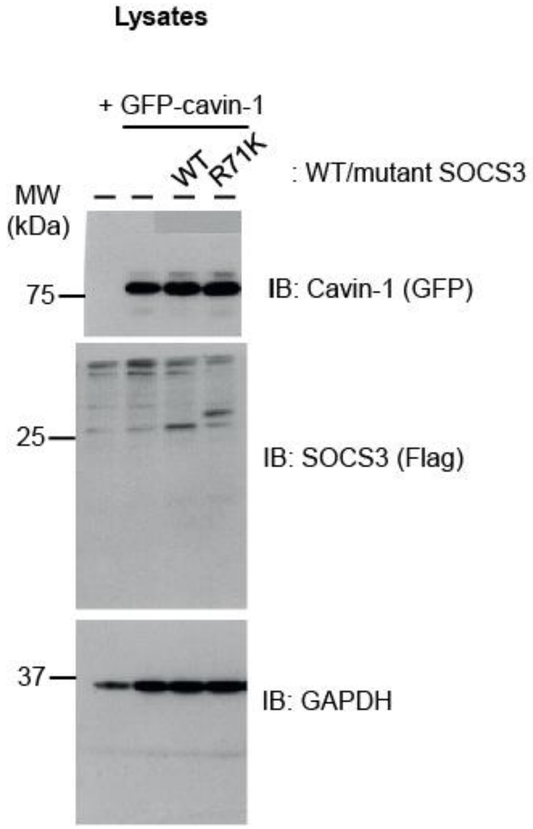
Lysates
+ GFP-cavin-1
WT
R71K
: WT/mutant SOCS3
MW (kDa)
75
IB: Cavin-1 (GFP)
25
IB: SOCS3 (Flag)
37
IB: GAPDH

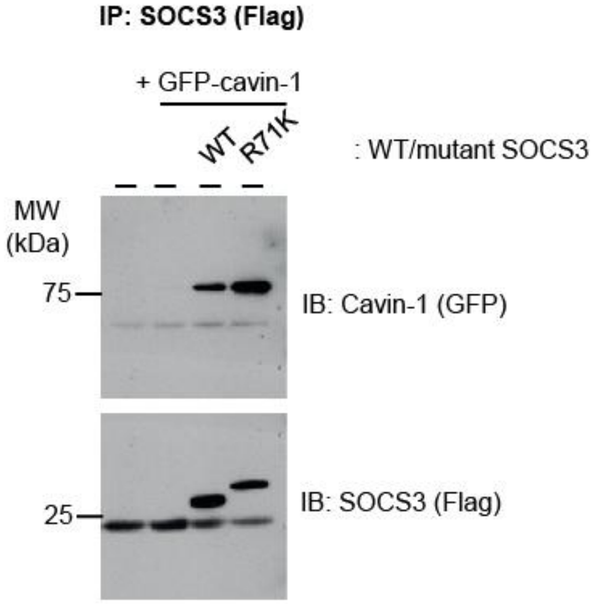
IP: SOCS3 (Flag)
+ GFP-cavin-1
WT
R71K
: WT/mutant SOCS3
MW (kDa)
75
IB: Cavin-1 (GFP)
25
IB: SOCS3 (Flag)

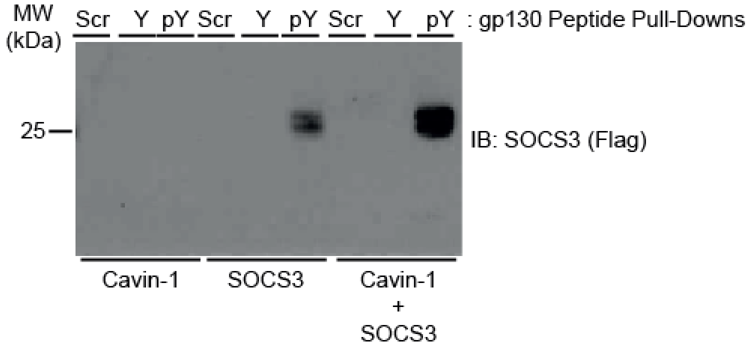
MW
(kDa)
Scr
Y
pY
Scr
Y
pY
Scr
Y
pY
: gp130 Peptide Pull-Downs
25
IB: SOCS3 (Flag)
Cavin-1
SOCS3
Cavin-1
+
SOCS3

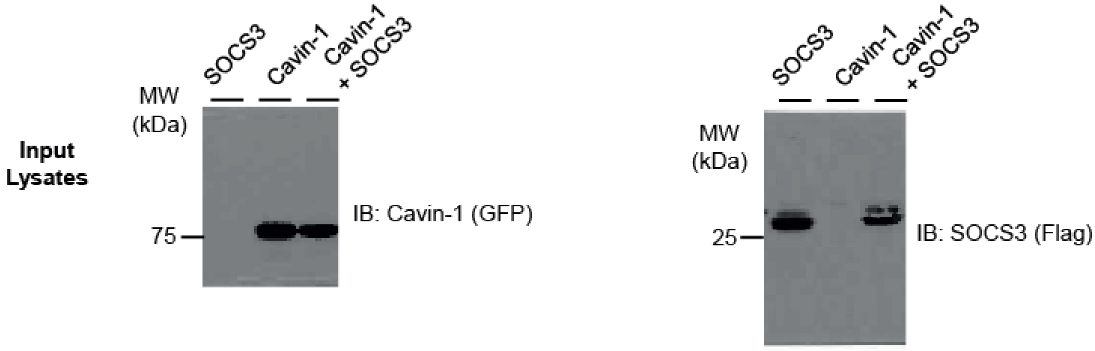
Input
Lysates
SOCS3
Cavin-1
Cavin-1
+ SOCS3
MW
(kDa)
75
IB: Cavin-1 (GFP)
SOCS3
Cavin-1
Cavin-1
+ SOCS3
MW
(kDa)
25
IB: SOCS3 (Flag)

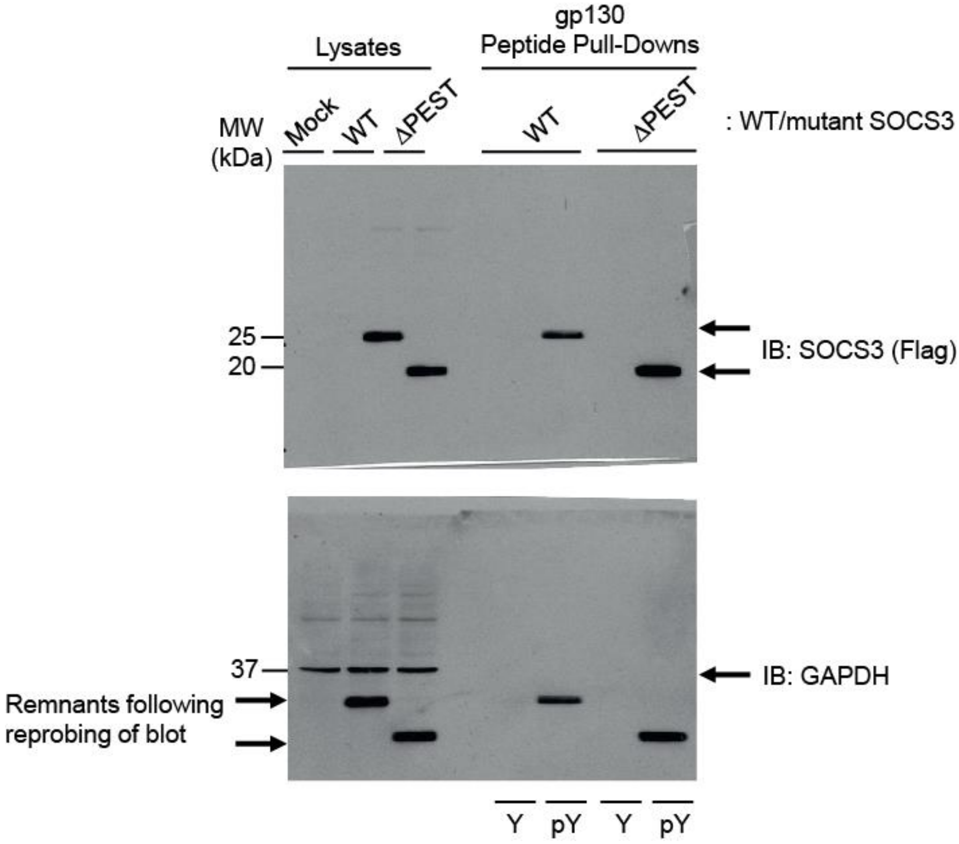
Lysates
gp130
Peptide Pull-Downs
MW
(kDa)
Mock
WT
ΔPEST
WT
ΔPEST
: WT/mutant SOCS3
25
20
IB: SOCS3 (Flag)
37
IB: GAPDH
Remnants following
reprobing of blot
Y
pY
Y
pY

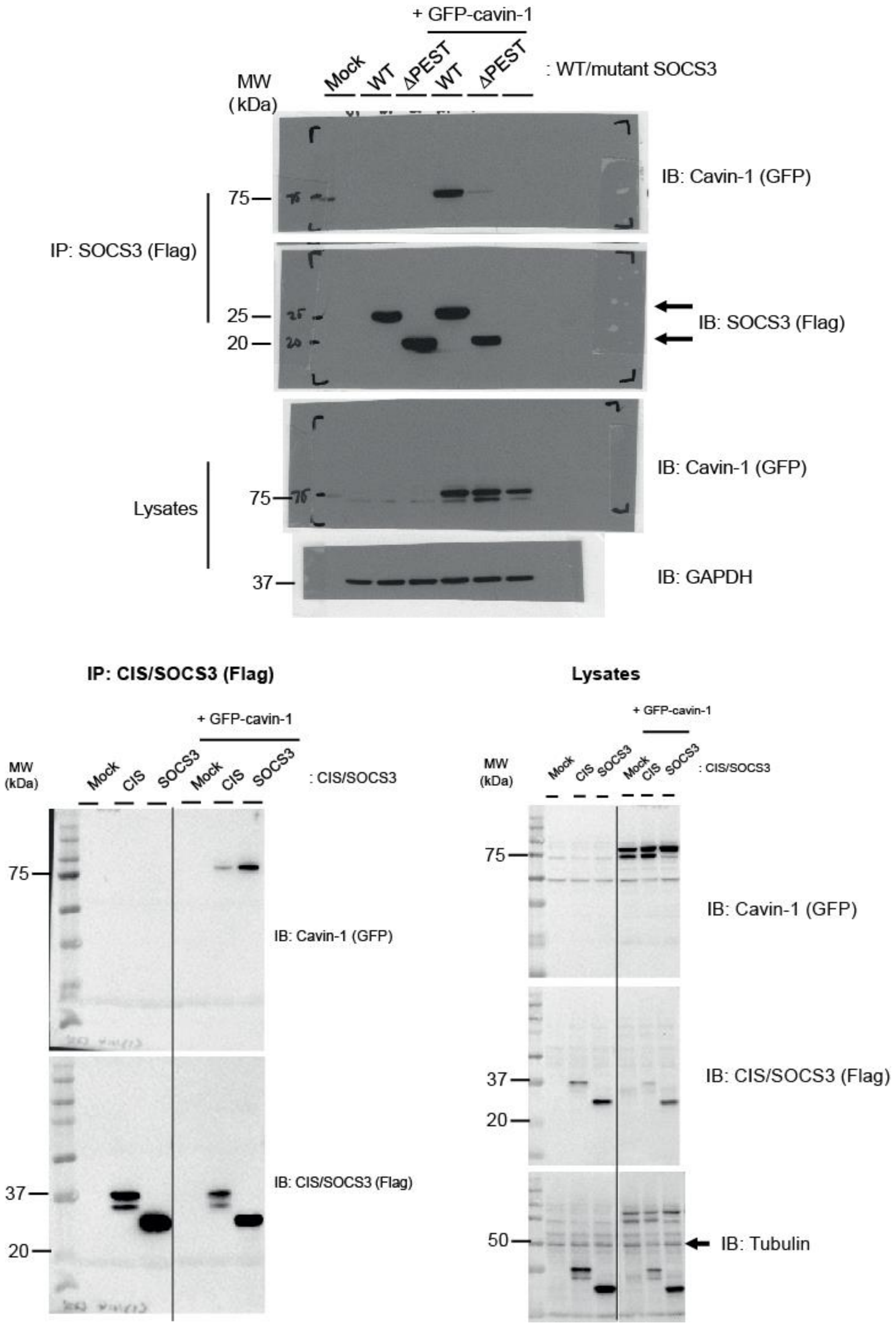

+ GFP-cavin-1
Mock
WT
ΔPEST
WT
ΔPEST
: WT/mutant SOCS3
MW (kDa)
IP: SOCS3 (Flag)
IB: Cavin-1 (GFP)
IB: SOCS3 (Flag)
Lysates
IB: Cavin-1 (GFP)
IB: GAPDH
75
25
20
75
37
IP: CIS/SOCS3 (Flag)
Lysates
+ GFP-cavin-1
Mock
CIS
SOCS3
Mock
CIS
SOCS3
: CIS/SOCS3
IB: Cavin-1 (GFP)
IB: CIS/SOCS3 (Flag)
IB: Tubulin
50

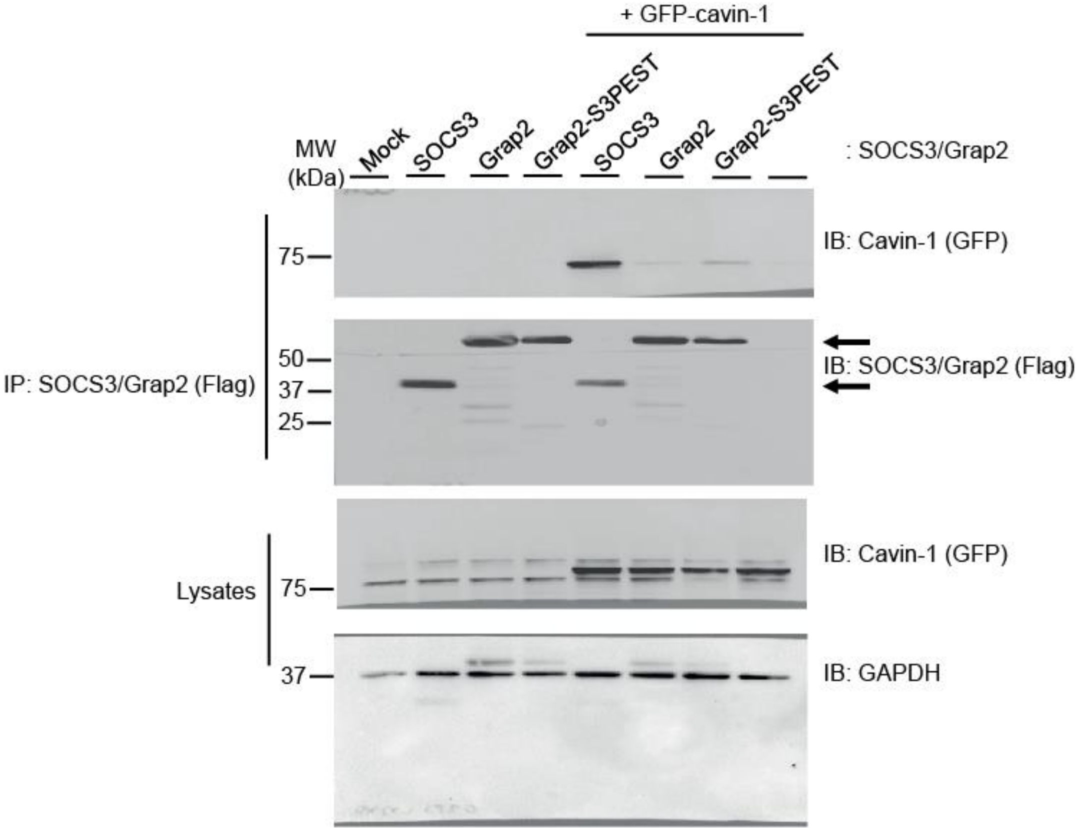
+ GFP-cavin-1
MW (kDa)
Mock
SOCS3
Grap2
Grap2-S3PEST
SOCS3
Grap2
Grap2-S3PEST
: SOCS3/Grap2
75
IB: Cavin-1 (GFP)
50
37
25
IP: SOCS3/Grap2 (Flag)
IB: SOCS3/Grap2 (Flag)
Lysates
75
IB: Cavin-1 (GFP)
37
IB: GAPDH

SOCS3

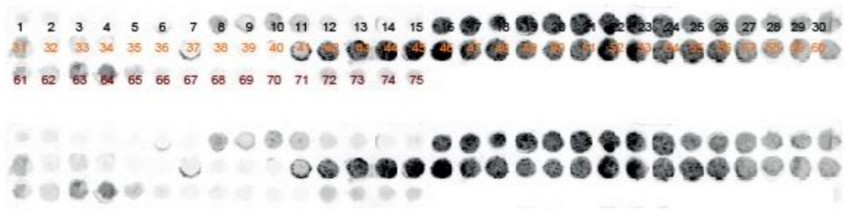

Cntrl

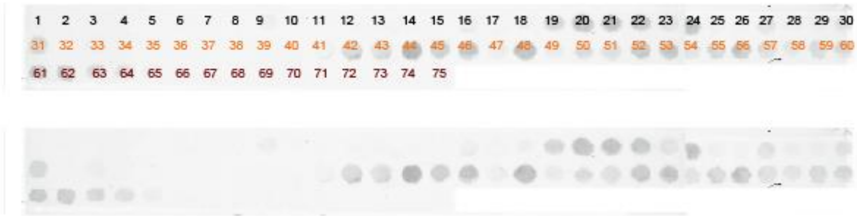
1 2 3 4 5 6 7 8 9 10 11 12 13 14 15 16 17 18 19 20 21 22 23 24 25 26 27 28 29 30
31 32 33 34 35 36 37 38 39 40 41 42 43 44 45 46 47 48 49 50 51 52 53 54 55 56 57 58 59 60
61 62 63 64 65 66 67 68 69 70 71 72 73 74 75

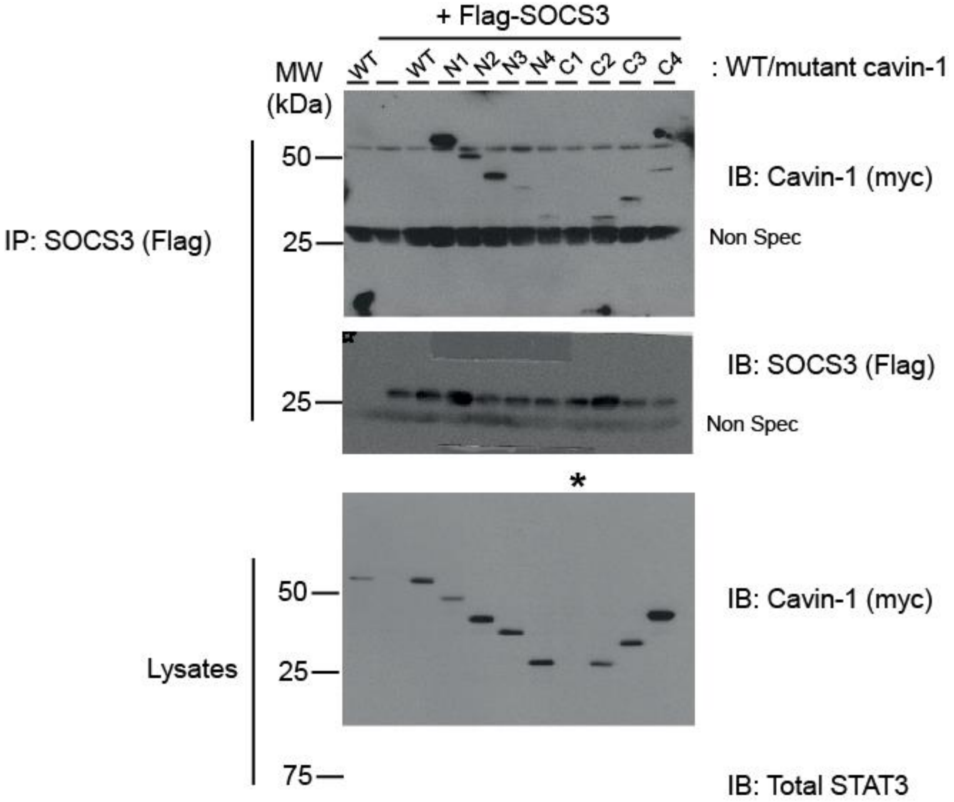
+ Flag-SOCS3
MW (kDa)
WT
WT
N1
N2
N3
N4
C1
C2
C3
C4
: WT/mutant cavin-1
IP: SOCS3 (Flag)
50
25
IB: Cavin-1 (myc)
Non Spec
25
IB: SOCS3 (Flag)
Non Spec
*
Lysates
50
25
IB: Cavin-1 (myc)
75
IB: Total STAT3

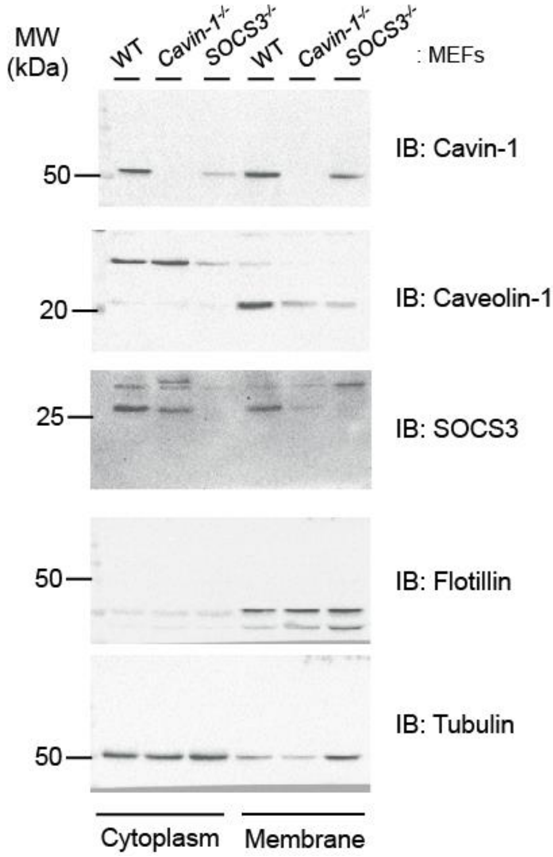
MW (kDa)
WT
Cavin-1-/-
SOCS3-/-
WT
Cavin-1-/-
SOCS3-/-
: MEFs
50
IB: Cavin-1
20
IB: Caveolin-1
25
IB: SOCS3
50
IB: Flotillin
50
IB: Tubulin
Cytoplasm
Membrane

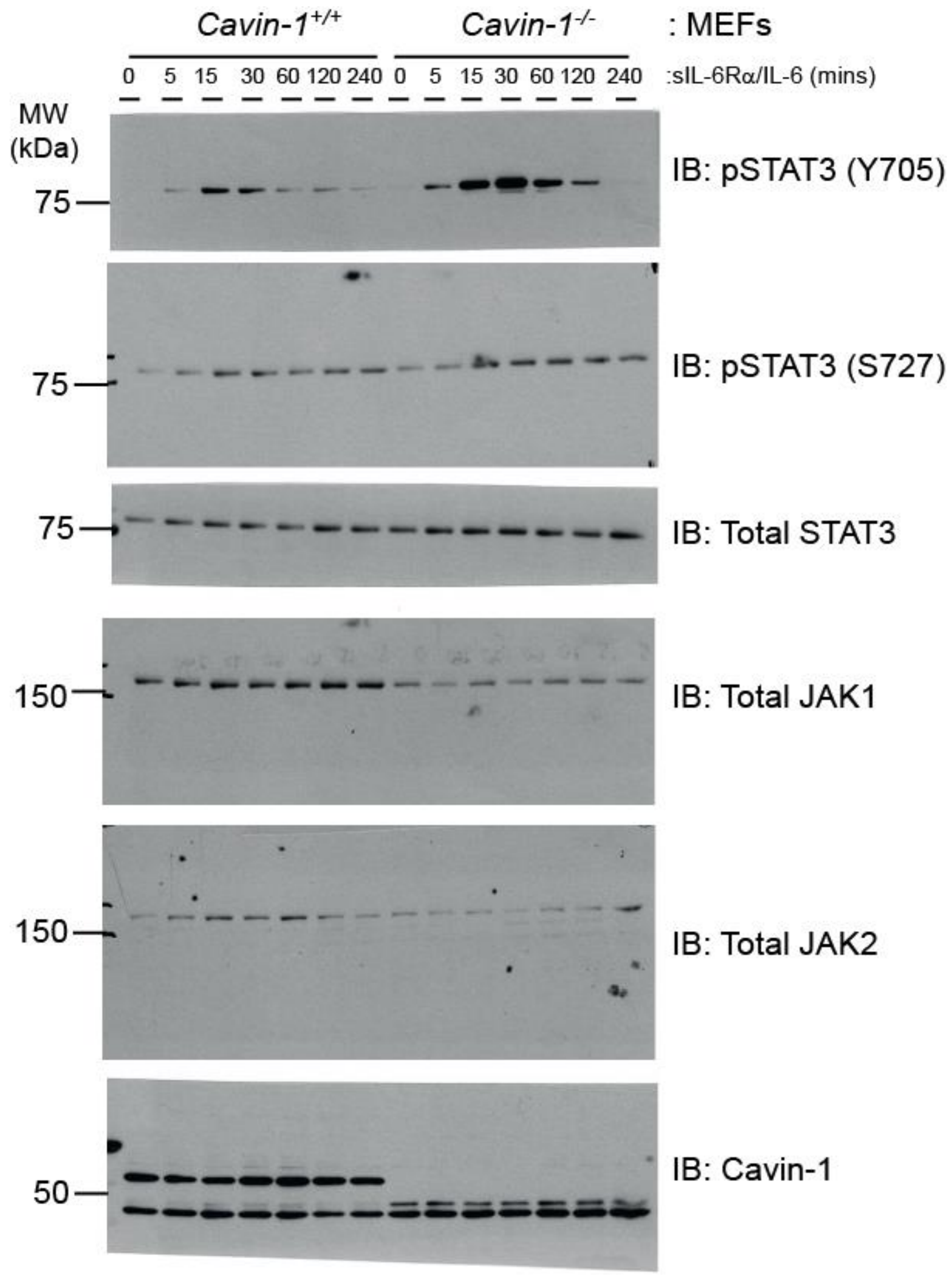

Cavin-1+/+
Cavin-1-/-
: MEFs
0 5 15 30 60 120 240 0 5 15 30 60 120 240
:sIL-6Rα/IL-6 (mins)
MW (kDa)
75
IB: pSTAT3 (Y705)
75
IB: pSTAT3 (S727)
75
IB: Total STAT3
150
IB: Total JAK1
150
IB: Total JAK2
50
IB: Cavin-1

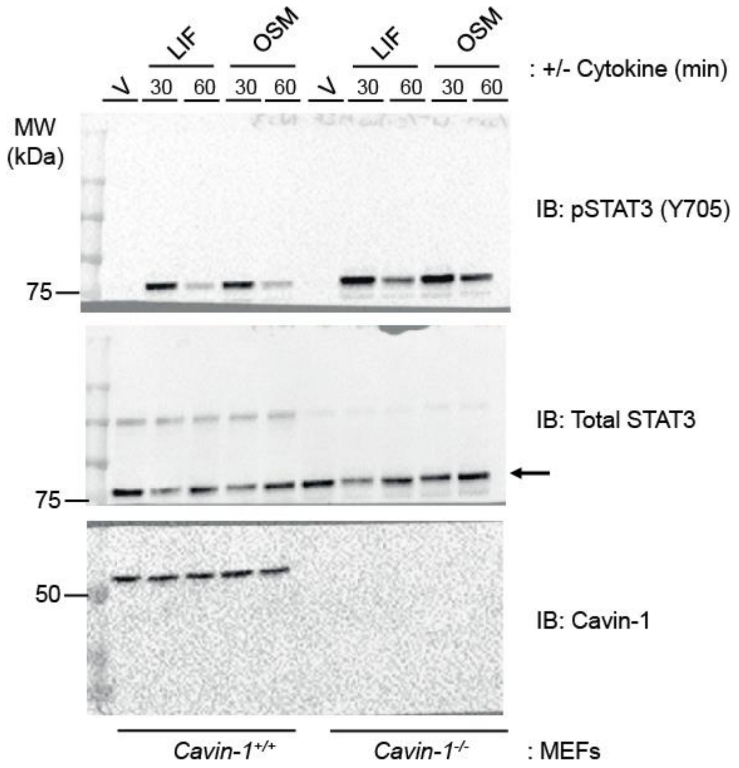

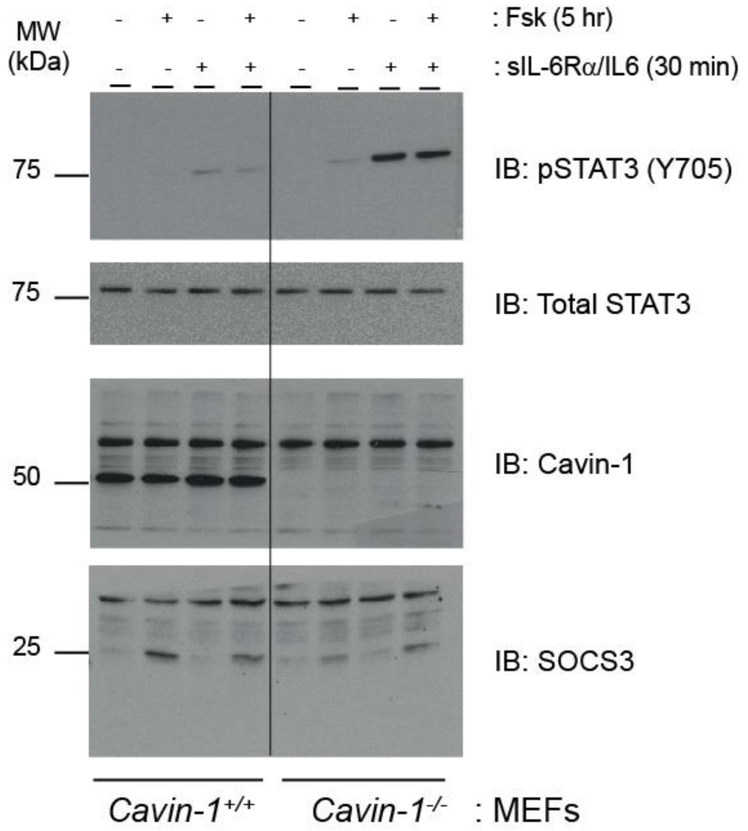

Supplementary Figure 7:

Uncropped blots used to generate data presented in Figures 1-9.
